# Ambulatory management of primary spontaneous pneumothorax: an open-label, randomised controlled trial

**DOI:** 10.1016/S0140-6736(20)31043-6

**Published:** 2020-07-04

**Authors:** Rob J Hallifax, Edward McKeown, Parthipan Sivakumar, Ian Fairbairn, Christy Peter, Andrew Leitch, Matthew Knight, Andrew Stanton, Asim Ijaz, Stefan Marciniak, James Cameron, Amrithraj Bhatta, Kevin G Blyth, Raja Reddy, Marie-Clare Harris, Nadeem Maddekar, Steven Walker, Alex West, Magda Laskawiec-Szkonter, John P Corcoran, Stephen Gerry, Corran Roberts, John E Harvey, Nick Maskell, Robert F Miller, Najib M Rahman

**Affiliations:** a Oxford Centre for Respiratory Medicine, University of Oxford, Oxford, UK; b Oxford Respiratory Trials Unit, University of Oxford, Oxford, UK; c Royal Berkshire National Health Service (NHS) Foundation Trust, Reading, UK; d Guy's and St Thomas' NHS Foundation Trust, London, UK; e Queen Margaret Hospital, NHS Fife, Dunfermline, UK; f Royal United Hospitals Bath NHS Foundation Trust, Bath, UK; g Western General Hospital, NHS Lothian, Edinburgh, UK; h West Hertfordshire Hospitals NHS Trust, Watford, UK; i Great Western Hospital NHS Foundation Trust, Swindon, UK; j University Hospitals of Morecambe Bay NHS Foundation Trust, Lancaster, UK; k Department of Medicine, University of Cambridge, Cambridge, UK; l North Bristol NHS Trust, Bristol, UK; m Blackpool Fylde and Wyre Hospitals NHS Foundation Trust, Blackpool, UK; n Queen Elizabeth University Hospital, Glasgow, UK; o Institute of Cancer Sciences, University of Glasgow, Glasgow, UK; p Kettering General Hospital, Kettering, UK; q Royal Infirmary of Edinburgh, NHS Lothian, Edinburgh, UK; r University Hospitals of North Midlands, Stoke-on-Trent, UK; s Academic Respiratory Unit, University of Bristol, Bristol, UK; t University Hospitals Plymouth NHS Trust, Plymouth, UK; u Centre for Statistics in Medicine, Nuffield Department of Orthopaedics, Rheumatology and Musculoskeletal Sciences, University of Oxford, Oxford, UK; v Institute for Global Health, University College London, London, UK

## Abstract

**Background:**

Primary spontaneous pneumothorax occurs in otherwise healthy young patients. Optimal management is not defined and often results in prolonged hospitalisation. Data on efficacy of ambulatory options are poor. We aimed to describe the duration of hospitalisation and safety of ambulatory management compared with standard care.

**Methods:**

In this open-label, randomised controlled trial, adults (aged 16–55 years) with symptomatic primary spontaneous pneumothorax were recruited from 24 UK hospitals during a period of 3 years. Patients were randomly assigned (1:1) to treatment with either an ambulatory device or standard guideline-based management (aspiration, standard chest tube insertion, or both). The primary outcome was total length of hospital stay including re-admission up to 30 days after randomisation. Patients with available data were included in the primary analysis and all assigned patients were included in the safety analysis. The trial was prospectively registered with the International Standard Randomised Clinical Trials Number, ISRCTN79151659.

**Findings:**

Of 776 patients screened between July, 2015, and March, 2019, 236 (30%) were randomly assigned to ambulatory care (n=117) and standard care (n=119). At day 30, the median hospitalisation was significantly shorter in the 114 patients with available data who received ambulatory treatment (0 days [IQR 0–3]) than in the 113 with available data who received standard care (4 days [IQR 0–8]; p<0·0001; median difference 2 days [95% CI 1–3]). 110 (47%) of 236 patients had adverse events, including 64 (55%) of 117 patients in the ambulatory care arm and 46 (39%) of 119 in the standard care arm. All 14 serious adverse events occurred in patients who received ambulatory care, eight (57%) of which were related to the intervention, including an enlarging pneumothorax, asymptomatic pulmonary oedema, and the device malfunctioning, leaking, or dislodging.

**Interpretation:**

Ambulatory management of primary spontaneous pneumothorax significantly reduced the duration of hospitalisation including re-admissions in the first 30 days, but at the expense of increased adverse events. This data suggests that primary spontaneous pneumothorax can be managed for outpatients, using ambulatory devices in those who require intervention.

**Funding:**

UK National Institute for Health Research.

## Introduction

Pneumothorax is a common clinical problem. Primary spontaneous pneumothorax describes patients developing pneumothorax in the absence of trauma, with no underlying established lung pathology, and occurs in in approximately 3000 patients per year in the UK.^1^, ^2^ Some patients can be managed conservatively with close observation,^3^, ^4^ but many symptomatic patients require an intervention to re-expand the lung. Current British Thoracic Society (BTS) guidelines suggest that aspiration of trapped air using a cannula and syringe should be considered, but more than 50% of patients subsequently require the insertion of a chest tube, which is then attached to an underwater seal drainage system.^4^ The mean duration of hospitalisation of patients admitted for drainage is 6–8 days.^5^

Ambulatory management of patients with primary spontaneous pneumothorax potentially removes the need for long hospital admission by facilitating outpatient treatment. Reducing the need for chest tubes with bulky underwater systems might allow patients to remain mobile and facilitate early discharge with the device in situ. Ambulatory devices generally incorporate a Heimlich (one-way) valve, which replaces the traditional underwater seal, either by in-line attachment to a standard chest tube or as part of an integrated device. However, the efficacy and safety of this approach has yet to be rigorously assessed. A systematic review^6^ of the literature found that 18 studies describing ambulatory management of both spontaneous and iatrogenic pneumothorax reported an overall success rate of 86% and successful outpatient management in 78% of cases, with few complications. Unfortunately, the available evidence was described as poor quality with a high risk of bias, including two inadequately powered randomised trials and several retrospective case series.^6^

**Research in context**

**Evidence before this study**

A search of PubMed and MEDLINE for articles on ambulatory management of pneumothorax published up to Dec 5, 2019, with the keywords “pneumothorax”, “ambulatory”, “Heimlich valve”, and “outpatient” showed that most studies were case series and included two small, inadequately powered randomised trials. In 2013, a systematic review of 18 studies found that although ambulatory options might be successful, the data were of poor quality with a high risk of bias. A further three case series were subsequently published. Given this uncertain evidence base, we designed the Randomised Ambulatory Management of Primary Pneumothorax trial as a controlled study to determine whether ambulatory management of patients with primary spontaneous pneumothorax reduces the length of hospitalisation and is safe.

**Added value of this study**

This study, which to our knowledge is the only large randomised trial of its kind, found that ambulatory management of patients with primary spontaneous pneumothorax significantly reduces the length of hospitalisation compared with standard care. Ambulatory patients had fewer pleural procedures, but serious adverse events were higher because of hospital re-admission.

**Implications of all the available evidence**

This trial challenges the current guidelines for management for patients with primary spontaneous pneumothorax, with the proviso that some patients will require further treatment.

The Randomised Ambulatory Management of Primary Pneumothorax (RAMPP) trial was done to determine whether ambulatory management of patients with primary spontaneous pneumothorax is safe and reduces the length of hospitalisation.

## Methods

### Study design

The RAMPP trial was a multicentre, open-label, randomised controlled trial comparing ambulatory management of primary spontaneous pneumothorax with standard care based on national guidelines.^4^ Participants were screened and recruited from 24 hospitals in the UK with a track record of recruiting to trials and a strong link between emergency and respiratory departments. Study oversight was provided by the trial steering committee and an independent data monitoring committee, and ethical approval was provided by the UK National Research Ethics Service Committee (15/SC/0240). The trial protocol can be found online and in the appendix (pp 9–47).^7^

### Participants

Eligible patients had presented with a symptomatic spontaneous pneumothorax (confirmed by a chest radiograph or CT scan) and were aged 16–55 years (as per BTS definition). Once a patient was identified as having a primary spontaneous pneumothorax, the decision to intervene was made on the basis of current BTS guidelines: patients were enrolled if they had a large pneumothorax (≥2 cm interpleural distance at the level of the hilum) or significant symptoms, or both. Patients were ineligible if they had known or suspected underlying lung disease (including >20 pack-year tobacco smoking history, but excluding well controlled asthma), evidence of tension pneumothorax (defined as clinical or radiographic evidence of significantly increased intrapleural pressure causing haemodynamic compromise that requires urgent decompression), or a contraindication to thoracic procedure as determined by the responsible physician, or if they were pregnant or lactating. Patients requiring intervention could be enrolled and randomly assigned up to 24 h after presentation, provided that they remained hospitalised with an ongoing symptomatic pneumothorax despite initial intervention (eg, patients treated initially with aspiration and observed overnight) requiring chest tube insertion. Patients not requiring an intervention were invited to participate in an observational cohort up to 2 weeks after their initial presentation, the results of which will be published separately. All participants in the current study provided written informed consent.

### Randomisation and masking

Patients were randomly assigned (1:1) to either insertion of an ambulatory device (Rocket Pleural Vent, Rocket Medical, Watford, UK) or standard care (aspiration, standard chest tube insertion, or both) as per BTS guidelines.^4^ Randomisation was done through a centralised, web-based system using a computer-generated minimisation algorithm. The minimisation factors were the recruiting centre and size of pneumothorax at presentation (≥4 cm *vs* <4 cm at the level of the hilum on chest radiograph). The study was necessarily open label, with both patients and physicians aware of treatment allocation.

### Procedures

Patients in the experimental arm had an ambulatory device inserted using sterile technique with up to 3 mg/kg lidocaine as local anaesthetic. The device was inserted either in the anterior mid-clavicular line (second intercostal space) or mid-axillary line (fifth intercostal space) according to investigator preference and clinical requirement. The ambulatory device used was an 8F gauge catheter attached to a self-contained, one-way Heimlich valve and fluid collection chamber (appendix p 1). Researchers and local clinicians were trained to insert the device by the trial coordinator or local Principal Investigator. Following device insertion, patients were observed for 1–2 h to assess them for clinical stability, after which a chest radiograph was done (appendix p 44).

If the chest radiograph showed insufficient lung re-expansion, the ambulatory device remained in situ and the patient was discharged, as long as the prespecified criteria of fitness for discharge were fulfilled. These criteria consisted of all of the following: the patient agreed, had clinically stable cardiorespiratory observations, had no increase in the size of pneumothorax since the last radiograph or review, did not require oxygen or other respiratory support, was mobile and able to self-care, was provided with written information on point of contact and follow-up plan, and lives with a responsible person at home. Any patient not meeting these criteria remained hospitalised and was reviewed daily. Sufficient lung re-expansion was defined as complete or almost complete re-expansion (<1 cm rim of air apically on chest radiograph) and being unable to aspirate air through the device using a connector and syringe.

If discharged home with the device in situ, patients were reviewed as an outpatient every 1–2 days (generally daily, but a single review during the weekend was sufficient if clinically stable) until day 4, at which stage the patient should be considered for thoracic surgery. The timing of the ongoing outpatient review after day 4 was at the discretion of the responsible clinician. After each review, if there was sufficient re-expansion of the lung and no ongoing air leak, the device was removed and the patient was discharged. A postremoval chest radiograph was done to ensure that the lung had not recollapsed. If the chest radiograph showed recurrence of pneumothorax, the patient was rehospitalised for observation and consideration of the placement of a standard chest tube connected to an underwater seal.

Patients who were in the control arm received standard treatment as per the 2010 BTS Pleural Guidelines.^4^ In brief, pleural aspiration (if the clinician deemed it appropriate) was attempted under local anaesthetic using a 14–16 gauge cannula and syringe, aspirating a maximum of 2·5 L. The patient was observed for 1–2 h to assess for clinical stability, and a repeat chest radiograph was done. If the repeat chest radiograph showed sufficient lung re-expansion, the patient was discharged. If the lung had not sufficiently re-expanded, then a small-bore chest tube (≤14 F) was inserted and attached to an underwater seal, and the patient was admitted to hospital.

Although initial aspiration was recommended in the trial protocol, the responsible clinician was able to proceed directly to chest tube insertion and admission at their discretion, in line with BTS guidelines.^4^ Decisions regarding chest tube removal were as per standard practice at participating centres but included no further air leak (as shown by a non-bubbling chest tube) and full lung expansion on chest radiograph. A postremoval chest radiograph was done to ensure that the lung had not recollapsed. Fitness for discharge criteria were assessed daily to provide equality between treatment groups. Evidence for the use of suction in patients with pneumothorax is poor. BTS guidelines suggest that high volume, low pressure suction should not be routinely used but could be considered if there is prolonged air leak.^4^ Use of suction was at the discretion of the local responsible clinician.

Patients were followed up at 1 week after the completion of treatment, and 30 days, 6 months, and 12 months after randomisation. Hospital admissions and pneumothorax recurrence were measured up to the day 30 follow-up point, and at each follow-up point, data were collected on further hospital admissions, pain and breathlessness visual analogue scale (VAS) scores, quality of life, and smoking status. Patients who were not able to attend face-to-face follow-up appointments were contacted by telephone to check for complications or recurrence, and if this was not possible, data on recurrence was collected through hospital or general practitioner medical records. Data on pneumothorax recurrence at 6 and 12 months will be presented separately with data from the observational cohort.

### Outcomes

The primary outcome was the total length of hospital stay up to 30 days after randomisation, including initial hospital stay and re-admissions. Patients remaining in hospital overnight were recorded as having a 1-day stay; those discharged on the same day (either after successful needle aspiration treatment or discharge with the ambulatory device) were recorded as zero length of stay. The 30-day point was chosen on the basis of previous data suggesting that most conservatively treated (non-surgical) primary spontaneous pneumothorax air leaks resolve within 14 days of initial treatment,^8^ and thus outcomes measured at 30 days were considered reliably to capture all related re-admissions. Patients with missing data at 1 week after treatment or 30 days after randomisation were assumed to have no re-admissions. Re-admission was defined as the need for emergency (non-planned) admission to hospital for any reason in relation to pneumothorax but was not restricted to the requirement for a further pleural intervention. Any re-admission to hospital was recorded as at least a 1-day stay. Planned day case reviews for outpatient treated patients were not included.

Secondary outcomes included the need for a further pleural procedure, adverse events, pain and breathlessness VAS scores, recurrence rates, and time off work because of pneumothorax treatment. Further pleural procedures were defined as any intervention that punctured the pleura (eg, chest drain insertion) after a first treatment intervention. We obtained a daily record of thoracic pain and breathlessness scores using a VAS score up to day 4 after randomisation, which was measured on a scale of 0 to 100 mm. A score of 0 indicated the complete absence of symptoms and 100 was the maximum possible level of symptoms, which is validated for assessment of breathlessness in patients having pleural procedures.^9^, ^10^ VAS scores were also recorded on completion of treatment. Because patients could be discharged with a small residual pneumothorax, those with a persistent small pneumothorax at 1-week follow-up were defined as having ongoing pneumothorax rather than a new occurrence. The recurrence of pneumothorax was defined as a new episode of symptomatic pneumothorax after full resolution on chest radiograph, or occurring after the 1-week follow-up visit. Several other secondary outcomes including quality of life questionnaires (EuroQol-5D-5L), blood parameters, and digital air flow analysis are planned for separate publications.

Concerning the failure of medical treatment, there is no robust evidence on the optimal timing for surgical intervention in primary spontaneous pneumothorax after initial treatment in the presence of ongoing air leak. Current BTS guidelines suggest that cases of persistent air leak or non-re-expansion should be referred for surgery after 3–5 days.^4^ To achieve objective outcomes for this study, the following criteria were developed to ensure consistent practice in both groups, and were recorded in all cases. Referral for thoracic surgery occurred in the presence of all of the following: (1) persistent air leak on day 4 after insertion of chest tube (as measured by bubbling chest tube attached to an underwater seal) or evidence of ongoing air leak through the ambulatory device; (2) persistent pneumothorax on chest radiograph; (3) patient agreed to have surgery; and (4) no contraindication to thoracic surgery.

Safety data were collected for all patients at each trial visit regarding adverse events, with seriousness defined as per Good Clinical Practice guidelines.^11^ Expected adverse events were pain at the drainage site, minor haemorrhage not requiring specific intervention (eg, blood transfusion or surgery), subcutaneous emphysema, pleural infection, unintentional removal (falling out) of the Pleural Vent or chest tube, recurrence of pneumothorax or worsening of ongoing pneumothorax (if evidence of failure of full resolution following initial intervention), re-expansion pulmonary oedema, and the need for further (non-emergency) pleural procedures. Expected serious adverse events included tension pneumothorax occurring during treatment, blockage of chest tube with clinical consequences (eg, patient unwell or further procedure required), major intrathoracic haemorrhage requiring specific intervention (eg, blood transfusion), and any additional emergency pleural procedure as deemed necessary by the local investigator (eg, large bore chest tube insertion). Because the recurrence of pneumothorax is common and expected (occurring in about 33% of primary spontaneous pneumothorax cases within 1 year^1^, ^12^), recurrence was not reported as a serious adverse event. Adverse events were assessed locally, and, if they fulfilled the criteria for a serious adverse event,^13^ they were reported immediately (ie, within 24 h) to the Oxford Respiratory Trials Unit for review. All serious adverse events were followed until resolution.

### Statistical analysis

The statistical analysis was approved by the trial statisticians and steering committee before data analysis. The sample size for the primary outcome was determined to detect a difference in length of hospital stay of 2·3 days: from a mean of 4 days admission in patients receiving standard care to an expected mean of 1·7 days in patients receiving ambulatory care (standard deviation in both groups 6·0). The sample size calculation included a correction factor for non-normal data. These estimates assume that up to 50% of patients receiving standard care were expected to be successfully treated with aspiration alone (ie, zero-day admission). It was assumed, conservatively, that about 20% of patients in the ambulatory care arm would require re-admission. Based on these parameters, with 80% power, a 5% two-sided significance level, and a 10% attrition rate, the study required 236 patients. Previous pleural studies have shown an attrition rate for the primary outcome measure of less than 5%.^14^, ^15^, ^16^

The Mann-Whitney *U* test was used for the primary analysis. The median hospital stay (expected to be non-normally distributed) was calculated for each group, and the 95% confidence interval for median difference was calculated using an exact method. Pre-planned sensitivity analyses were done to assess the robustness of the primary outcome. Survival analysis techniques were used to compare time to discharge between the two groups using the Gehan-Breslow-Wilcoxon test (more appropriate than the log-rank test because of a high early event rate in primary spontaneous pneumothorax) and using Cox proportional hazards regression to calculate a hazard ratio and associated 95% confidence interval. An analysis of hospital stay in hours (instead of whole days) was done using identical methods to the primary analysis. The Student's *t* test was used to compare mean differences in length of stay. A worst-case scenario sensitivity analysis of the primary outcome was done by assigning the longest possible initial hospital day to those patients who were missing follow-up forms in the ambulatory care arm.

Continuous secondary outcome measures were analysed using ANCOVA adjusting for baseline scores. The adjusted mean difference between treatment arms was calculated with 95% confidence interval and p values. Categorical secondary outcome measures were analysed using the χ^2^ test. Time-to-event secondary outcome measures were analysed in the same way as time-to-event analysis of the primary outcome measure.

Prespecified subgroup analyses of the primary outcome were done for gender, previous pneumothorax, and tobacco and marijuana smoking history (ever *vs* never), using tests of interaction in Cox regression models for the primary outcome. All results are reported in concordance with CONSORT standards.^17^ All analyses were done using Stata (version 15, StataCorp 2017, USA).

The trial was prospectively registered with the International Standard Randomised Clinical Trials Number ISRCTN79151659.

### Role of the funding source

Neither of the funding sources were involved in the study design, data collection, data analysis, data interpretation, or writing of the report. The corresponding author had full access to all the data in the study and had final responsibility for the decision to submit for publication.

## Results

**Figure 1:**
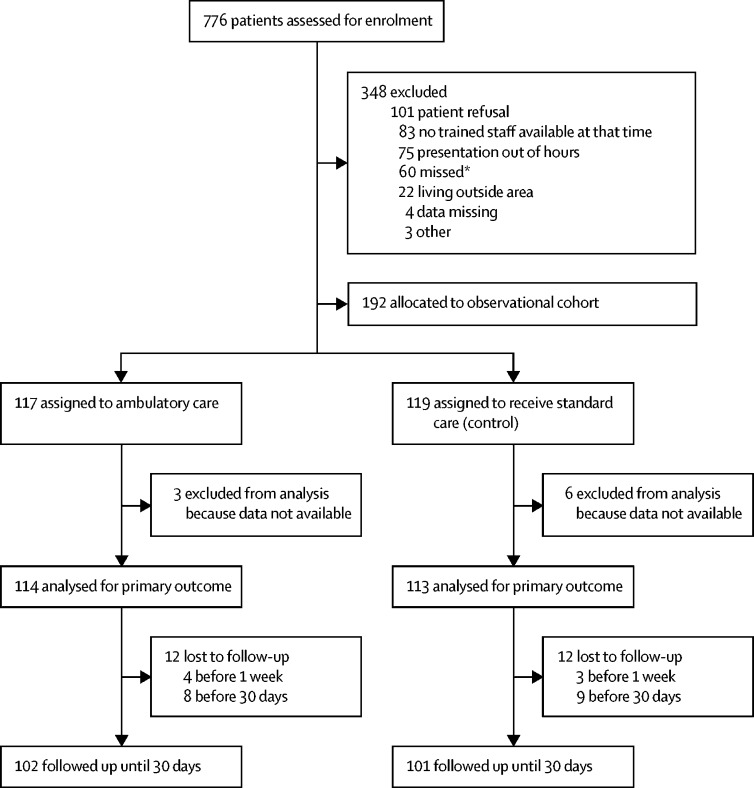
Trial profile *Patients were classed as missed if they had already been treated for primary spontaneous pneumothorax and were therefore no longer eligible.

We recruited patients between August 27, 2015, and March 12, 2019. After approaching 776 potentially eligible patients, we reached our target of 236 (30%) participants, of whom 117 were assigned to ambulatory care and 119 to standard care (figure 1). The mean age at recruitment was 30 years (SD 8). 193 (82%) patients were male, 58 (25%) had a history of previous pneumothorax, and 20 (8%) had a family history (first-degree or second-degree relative) of pneumothorax (table 1). 161 (68%) patients were current or former tobacco smokers, with a median pack-year history for both groups combined of 8 (IQR 5–12), and 114 (48%) were current or former marijuana smokers (table 1). Most patients were symptomatic with either chest pain (213 [90%]) or shortness of breath (210 [89%]).

**Table 1:** Baseline characteristics by treatment allocation

		**Patients receiving ambulatory care (n=117)**	**Patients receiving standard care (n=119)**
Gender			
	Female	21 (18%)	22 (18%)
	Male	96 (82%)	97 (82%)
Age (mean [SD])		31 (8)	30 (9)
Ethnicity			
	Asian	2 (2%)	5 (4%)
	Black, African, or Caribbean	1 (1%)	3 (3%)
	Mixed or multiple ethnicity	1 (1%)	1 (1%)
	Other	1 (1%)	2 (2%)
	Unknown	1 (1%)	1 (1%)
	White	111 (95%)	107 (90%)
Hours of pneumothorax before admission*			
	Median (IQR)	15 (4–72); n=105	23 (5–48); n=112
	Mean (SD)	59 (132); n=105	58 (147); n=112
Size of pneumothorax			
	<4 cm	49 (42%)	49 (41%)
	≥4 cm	68 (58%)	70 (59%)
Side of pneumothorax			
	Right	63 (54%)	68 (57%)
	Left	54 (46%)	51 (43%)
Chest pain			
	Yes	104 (89%)	109 (92%)
	No	13 (11%)	10 (8%)
Shortness of breath			
	Yes	104 (89%)	106 (89%)
	No	13 (11%)	13 (11%)
Previous procedure to treat pneumothorax			
	No	96 (82%)	106 (89%)
	Yes	21 (18%)	13 (11%)
Previous pneumothorax			
	No	90 (77%)	87 (73%)
	Yes	26 (22%)	32 (27%)
	Missing data	1 (1%)	..
Previous other lung disease			
	No	109 (93%)	113 (95%)
	Yes	5 (4%)	6 (5%)
	Unknown	1 (1%)	..
	Missing data	2 (2%)	..
Cardiovascular disease			
	No	113 (97%)	100 (100%)
	Yes	3 (3%)	..
	Missing	1 (1%)	..
Renal disease			
	No	115 (98%)	117 (98%)
	Yes	1 (1%)	2 (2%)
	Missing	1 (1%)	..
Family history of pneumothorax			
	No	100 (85%)	100 (84%)
	Yes	7 (6%)	13 (11%)
	Unknown	8 (7%)	6 (5%)
	Missing	2 (2%)	..
Tobacco smoking status			
	Current smoker	61 (52%)	56 (47%)
	Ex-smoker	18 (15%)	26 (22%)
	Never smoked	35 (30%)	37 (31%)
	Missing	2 (2%)	..
	Unknown	1 (1%)	..
Marijuana smoking status			
	Never smoked	57 (49%)	55 (46%)
	Current smoker	35 (30%)	31 (26%)
	Ex-smoker	20 (17%)	28 (24%)
	Unknown	3 (3%)	5 (4%)
	Missing	2 (2%)	..
Tobacco smoking pack-years for current and previous smokers (median [IQR])		8 (5–12); n=74	7 (5–11); n=78

Data are n (%) unless otherwise indicated.

* Not all patients had available data.

**Figure 2:**
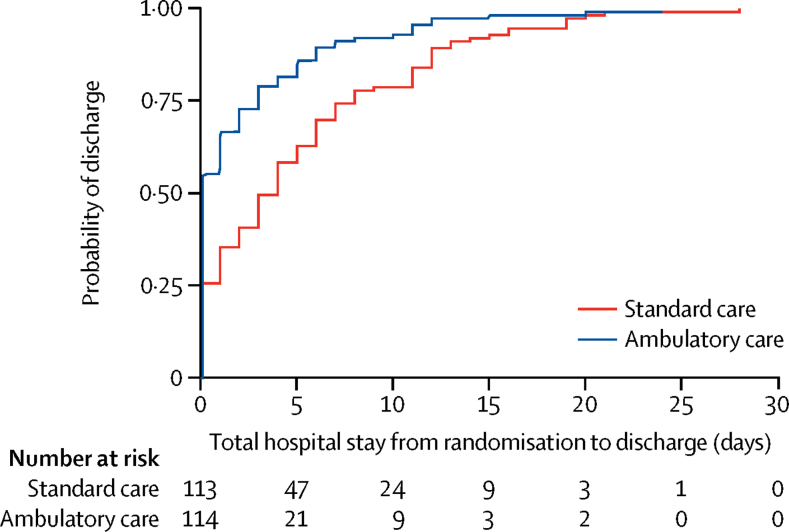
Cumulative incidence curve showing time to discharge from randomisation plus re-admissions within 30 days

For the primary outcome, we analysed 114 (97%) of 117 patients who received ambulatory care and 113 (95%) of 119 who received standard care, because data was not available for the remaining patients. Among those who had ambulatory care, the median hospital stay in the first 30 days was 0 days [IQR 0–3], which was significantly lower than in those who received standard care (4 days [IQR 0–8]; median difference 2 days, 95% CI 1–3; p<0·0001). The cumulative incidence of hospital stay (comprising any initial hospital stay plus re-admission days in hospital) is shown in figure 2. The difference in overall hospital stay during 30 days was also significant when calculated in hours (ambulatory median 4·7 h [IQR 2·7–59·2] *vs* standard care median 74·7 h [IQR 6·3–178·2); p<0·0001; table 2). In the ambulatory care group, 73 (64%) of 114 patients with available data were discharged on the same day as admission, compared with 39 (34%) of 115 patients with available data in the standard care group (p<0·0001; appendix p 3). In 24 patients (12 in ambulatory care; 12 in standard care), data were missing either at 1-week or at 30-days follow-up, or both.

**Table 2:** Primary outcome according to treatment arm

	**Patients receiving ambulatory care (n=117)**		**Patients receiving standard care (n=119)**		**Difference (95% CI)**	**p value**
	Patients with available data	Result	Patients with available data	Result		
Total hospital stay within 30 days after treatment (days)	114	0 (0 to 3)	113	4 (0 to 8)	2 (1 to 3)	<0·0001
Total hospital stay within 30 days after treatment (h)	114	4·7 (2·7 to 59·2)	110	74·7 (6·3 to 178·2)	48·3 (19·4 to 71·8)	<0·0001
Duration of initial hospital stay (days)	114	0 (0 to 1)	114	2 (0 to 6)	1 (0 to 2)	<0·0001
Patients requiring further admission within 30 days (n [%])	117	17 (15%)	119	23 (19%)	..	..
Duration of further admission (days)	17	4 (3 to 7)	23	4 (2 to 5)	−1 (−3 to 1)	0·285
Time until successful completion of treatment (days)	111	3 (1 to 6)	115	2 (0 to 6)	−1 (−2 to 0)	0·0040
Patients discharged on same day as initial admission (n [%])	114	73 (64%)	115	39 (34%)	..	<0·0001

Data are median (IQR) unless otherwise indicated.

Re-admission rates were similar in the ambulatory and standard care groups (table 2). The time until successful completion of treatment, defined as when the ambulatory device was removed (ambulatory care) or when the patient had a successful outcome from aspiration or chest tube removal after drainage (standard care), was significantly longer in patients in the ambulatory care group (table 2). The total time off work did not differ between groups, with a mean of 10·7 days (SD 11·9) in the ambulatory care group and 11·5 days (SD 13·0) in the standard care group.

The prespecified subgroups also showed differences in hospital stay, including for previous history of pneumothorax and smoking history (tobacco and marijuana; table 3). The tests for interactions were not significant in any of the subgroup analyses; hence, conclusions cannot be drawn about differential benefits in the subgroups.

**Table 3:** Primary outcome by prespecified subgroup analysis

		**Patients receiving ambulatory care (n=117)**		**Patients receiving standard care (n=119)**		**Median difference (95% CI)**	**p value**
		Patients with available data	Result	Patients with available data	Result		
Gender		..	..	..	..	..	0·518
	Female	21	2 (0–6)	20	5 (2–11)	2 (0–5)	..
	Male	93	0 (0–2)	94	3 (0–7)	2 (1–3)	..
Previous pneumothorax		..	..	..	..	..	0·445
	Yes	24	0 (0–5)	30	4 (0–8)	1 (0–5)	..
	No	89	0 (0–3)	84	4 (1–8)	2 (1–3)	..
Tobacco smoking history		..	..	..	..	..	0·209
	Ever	76	0 (0–3)	79	4 (1–7)	2 (1–4)	..
	Never	35	0 (0–2)	35	3 (0–11)	2 (0–5)	..
Marijuana smoking history		..	..	..	..	..	0·254
	Ever	53	0 (0–3)	56	4 (1–8)	3 (1–4)	..
	Never	56	0 (0–3)	53	3 (0–7)	2 (0–4)	..

Data are median (IQR) unless otherwise indicated. p value compares difference between subgroups.

In the worst-case scenario sensitivity analysis, median hospital stay in the first 30 days remained significantly shorter in those who received ambulatory care (1 day [IQR 0–6]) than in those who received standard care (4 days [IQR 0–8]; median difference 1 day, 95% CI 0–2; p=0·0057).

Because not all patients in the standard care arm had aspiration, we did a post-hoc analysis of the primary outcome excluding patients in whom no aspiration was done (ie, who proceeded directly to chest drain insertion as per clinician preference). Removing the 31 (26%) of 119 patients in whom no aspiration was attempted in the standard care arm, the primary outcome still favoured the intervention arm. The median length of stay for patients in the standard care arm in whom aspiration was done was 3 days (IQR 0–8), and the median difference was 1 (95% CI 0–3; p=0·0001).

Regarding the secondary outcomes, initial treatment data was available for 227 (96%) of 236 patients. Documented per-protocol compliance with allocated treatment was 114 (97%) of 117 patients receiving ambulatory care and 113 (95%) of 119 patients receiving standard care. Among 119 patients receiving standard care, initial management was pleural aspiration in 81 (68%) patients, 38 (47%) of whom subsequently required chest drain insertion. 31 (26%) of 119 patients receiving standard care were initially managed with a chest drain (table 4).

**Table 4:** Secondary outcomes: need for further procedures

		**Patients receiving ambulatory care (n=117)**		**Patients receiving standard care (n=119)**		**p value**
		Patients with available data	Result	Patients with available data	Result	
Initial management						
	Pleural Vent inserted	117	114 (97%)	119	0	..
	Aspiration	117	0	119	81 (68%)	..
	Chest tube	117	0	119	31 (26%)	..
	Other	117	0	119	1 (1%)	..
	Unknown*	117	3 (2%)	119	6 (5%)	..
Patients requiring additional procedure		114	24 (21%)	113	42 (35%)	0·0075
Additional procedure required						
	Further Pleural Vent replacement	114	2 (2%)	113	0	..
	Chest tube after aspiration	114	0	113	38 (32%)	..
	Further chest tube	114	13 (11%)	113	4 (3%)	..
	Larger chest tube	114	2 (2%)	113	1 (1%)	..
	Repeat aspiration	114	4 (3%)	113	3 (3%)	..
	Suction applied	11	1 (1%)	113	1 (1%)	
Total number of procedures per patient (mean [SD])		114	1·2 (0·5)	113	1·4 (0·7)	0·033

Data are n (%) unless otherwise indicated.

* Data not recorded on database.

Following the initial intervention, patients who received ambulatory care required fewer total pleural procedures (mean 1·2 [SD 0·5]) than did those who received standard care (mean 1·4 [SD 0·7]; p=0·0327), and 40 (33·6%) of 119 patients who received standard care required one or more further pleural procedures (table 4). Surgical referral rates were similar in both groups, including 33 (28%) of 117 patients who received ambulatory care and 26 (22%) of 119 patients who had standard care. Of patients referred for surgery, 21 (64%) in ambulatory care and 19 (73%) in standard care then had a surgical procedure.

**Figure 3:**
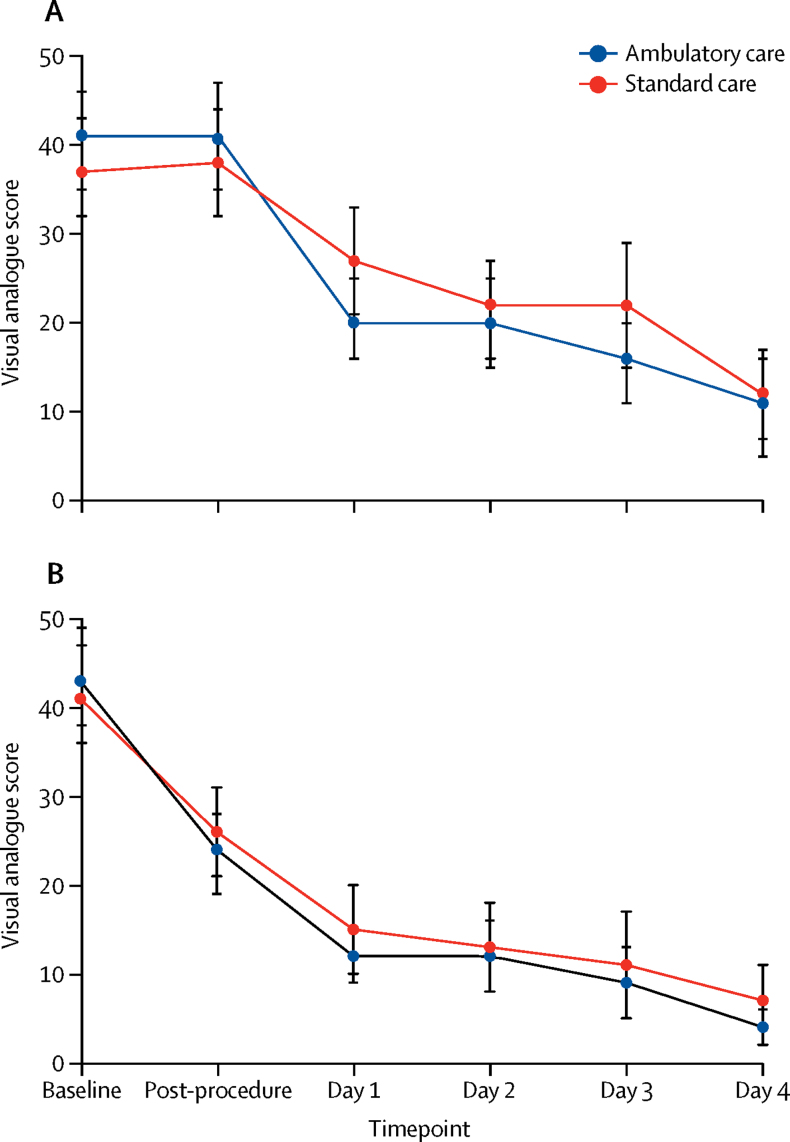
Visual Analogue Score of pain (A) and breathlessness (B) Scores shown for baseline (at enrolment), after the initial procedure, and then daily on days 1–4. Bars represent confidence intervals.

Mean pain and breathlessness VAS scores were high at baseline and improved with treatment in both treatment groups (figure 3). Reported pain after the first intervention was similar in both groups and improved during days 1–4. There was no significant difference in analgesia use between the two groups during days 0–4 (appendix pp 5–6).

Among the study population, 1 week after completion of treatment, 26 (12%) of 220 patients had an ongoing pneumothorax (not requiring admission) and 27 (12%) had a recurrence (appendix p 7). The proportion of patients with recurrence at day 7 was lower in the ambulatory care group (8 [7%] of 110 patients) than in the standard care group (19 [19%] of 110 patients; p=0·02). Of 19 patients receiving standard care with recurrence, 10 (53%) were initially treated with aspiration, five (26%) had aspiration then chest tube insertion, and four (21%) had chest tube insertion. A further 19 patients (9%) had a recurrence between day 7 and day 30.

Follow-up data were available up to 12 months for 181 (77%) of 236 patients, with the remainder lost to follow-up. Recurrence rates from 1–12 months were similar in the ambulatory and standard care arms (appendix p 7). The overall rate of ipsilateral pneumothorax recurrence up to 12 months was 24% for the ambulatory arm and 28% for the standard care arm (p=0·22; appendix p 8). Recurrence-free survival curves are shown in the appendix (p 4).

110 (47%) of 236 patients had adverse events (table 5), including 64 (55%) of 117 patients in the ambulatory care arm and 46 (39%) of 119 in the standard care arm. 97 (39%) patients had intervention-related or treatment-related adverse events.

**Table 5:** Adverse events

		**Patients receiving ambulatory care (n=117)**	**Patients receiving standard care (n=119)**	**p value**
Any serious adverse event or adverse event		64 (55%)	46 (39%)	0·0135
Serious adverse events		14 (12%)	0	<0·0001
Serious adverse events related to treatment*				
	Enlarging pneumothorax†	4 (3%)	0	..
	Device blocked or kinked†	2 (2%)	0	..
	Device dislodgement†	1 (1%)	0	..
	Re-expansion pulmonary oedema (asymptomatic)	1 (1%)	0	..
	Device leakage†	1 (1%)	0	..
	Admitted for suction	1 (1%)	0	..
Serious adverse events unrelated to treatment*				
	Unrecognised haemopneumothorax†	3 (3%)	0	..
	Pleurisy	1 (1%)	0	..
Adverse events related to treatment*		51 (44%)	40 (34%)	0·1154
	Pain at tube site	36 (31%)	36 (30%)	..
	Haematoma or bleeding	8 (7%)	2 (2%)	..
	Subcutaneous emphysema	7 (6%)	7 (6%)	..
	Site infection	1 (1%)	1 (1%)	..
	Tube displacement	2 (2%)	1 (1%)	..
	Drainage device failure	3 (3%)	1 (1%)	..
	Blocked tube	1 (1%)	1 (1%)	..
	Fluid within tube	3 (3%)	0	..
	Other chest pain	2 (2%)	4 (3%)	..
	Erythema or itch	2 (2%)	0	..
	Attendance at emergency department	1 (1%)	0	..
Adverse events not related to treatment		9 (8%)	10 (8%)	0·8410
	Chest infection	0	3 (3%)	..
	Infection at site	2 (2%)	1 (1%)	..
	Other chest pain	1 (1%)	1 (1%)	..
	Vasovagal episode	2 (2%)	0	..
	Shortness of breath	1 (1%)	1 (1%)	..
	Blood in pleural effusion	1 (1%)	0	..
	Pleural effusion	0	1 (1%)	..
	Erythema or itch	0	1 (1%)	..
	Fainting	1 (1%)	0	..
	Nausea and vomiting	0	1 (1%)	..
	Per rectum bleeding	1 (1%)	0	..
	Viral infection	0	1 (1%)	..
	Attendance at emergency department	1 (1%)	0	..

Data are n (%).

* Patients could have more than one adverse event.

† Required admission and chest tube insertion.

All 14 serious adverse events occurred in patients who received ambulatory care, and these events were all rated as serious on the basis of the need for re-admission to hospital (table 5). Eight (57%) of the 14 serious adverse events were related to the intervention (and some patients had more than one serious adverse event): four (3%) of 117 patients receiving ambulatory care had an enlarging pneumothorax on repeat chest radiography at outpatient review, which required admission and chest tube insertion; for two (2%) patients the device malfunctioned (tube kinked or blocked); one (1%) patient had radiographic evidence of (asymptomatic) pulmonary oedema at review on day 1, but was admitted for observation without overnight stay; one (1%) device was leaking fluid; the device dislodged in one (1%) patient, requiring chest tube placement; and one (1%) patient was admitted for suction. The remaining four serious adverse events were haemopneumothorax (n=3 [3%]) and pleurisy (n=1 [1%]), which were all judged to be unrelated to the intervention. All three patients with unrecognised haemopneumothorax had chest radiographic evidence of significant fluid (in addition to pneumothorax) before the intervention. These three patients either had surgery or CT angiography that did not identify an intervention-associated source of bleeding.

The most frequent non-serious adverse events were pain at the insertion site, haematoma or bleeding, surgical or subcutaneous emphysema, and failure of the drainage device (table 5).

## Discussion

This multicentre, open-label, randomised controlled trial compared an ambulatory treatment strategy for primary spontaneous pneumothorax with standard care using evidence-based guidelines (aspiration, chest drain insertion, or both), using clinically relevant outcomes including the duration of hospital stay during the first 30 days following intervention. The results show the efficacy of the ambulatory strategy, with most patients successfully managed as outpatients. This finding is supported by analysis of the primary outcome by initial hospital and overall stay in both whole days and number of hours.

Although 24 (21%) of 114 patients who had ambulatory care required at least one further procedure, patients in this group required significantly fewer procedures overall than those who received standard care. Patients in the ambulatory group had longer treatment duration (ie, time with ambulatory device in place) than did the standard care group (ie, time with chest drain in place). This difference is probably due to a number of factors. First, physicians might have been more cautious in managing patients with the ambulatory device and left the device in situ longer to ensure it was not removed too soon. Second, because patients in the ambulatory care group were being managed as outpatients and reviewed every 24 h, there were fewer opportunities to assess whether the pneumothorax had resolved than there were for those with a chest tube, who were treated in hospital and potentially reviewed many times per day.

Serious adverse events, defined as those needing admission to hospital, occurred exclusively in the ambulatory care arm. Serious adverse events related to treatment included enlargement of pneumothorax despite the ambulatory device being in place and device blockage and kinking, requiring chest tube insertion and hospitalisation. In the ambulatory care group, three patients had unrecognised haemopneumothoraces, which at review were not considered related to the intervention. Although this higher rate of serious adverse events is clearly important, by the nature of its definition it is biased against the ambulatory arm. Insertion of a chest tube (eg, after failed aspiration) and admission to hospital was not a possible serious adverse event in the standard care arm because this constituted normal care. Therefore, any adverse event in a patient already hospitalised, regardless of the primary intervention, requires careful interpretation. Nonetheless, the increased adverse event rate in the ambulatory arm should be accounted for when using an ambulatory pneumothorax strategy.

No significant differences between intervention groups were observed in secondary outcome measures in terms of VAS pain or breathlessness scores, time off work, and overall recurrence at 30 days. However, the pneumothorax recurrence rate within 7 days of treatment completion was higher in those who received standard care. Recurrence at this early stage is unusual and is often presumed to be due to enlargement of an ongoing pneumothorax. As a result, investigators classified patients with pneumothorax within 7 days into two categories: ongoing if there had been incomplete re-expansion on chest radiograph at completion of treatment, or new recurrence if the pneumothorax had fully resolved on the last chest radiograph. The excess recurrence in patients receiving standard care was not entirely due to patients treated with aspiration alone, because they accounted for less than 53% of those with early recurrence.

Longer-term recurrence rates (1–12 months) were similar between the two groups. Despite excess recurrence in the standard care arm by 30 days, the cumulative rate of first ipsilateral recurrence was not statistically different at 12 months. The overall rate of recurrence (27%) is consistent with previous large epidemiological studies.^1^, ^12^

A 2020 randomised trial^3^ in primary spontaneous pneumothorax compared conservative management (ie, no intervention) with standard care (ie, chest tube insertion),^3^ reporting that the conservative approach was non-inferior to standard care in the primary outcome of resolution of pneumothorax on chest radiograph at 8 weeks. We have concerns about the widespread adoption of this approach. First, the trial screened more than 2600 patients to randomly assign 316, and it required 39 sites recruiting for 6 years. Excluding patients with previous pneumothorax would automatically exclude about 25% of the population. The reasons for which all other patients were excluded in the trial are unclear. The trial might therefore be applicable only to those patients with minimal symptoms, and it is already recognised that conservative management is wholly appropriate in some cases of primary spontaneous pneumothorax (as stated in the 2010 BTS guidelines^4^). Second, the primary outcome was radiological, rather than patient-centred (eg, symptoms, time in hospital). Few data are presented on patient symptoms except at 2 weeks and what was termed an overall satisfaction score. Therefore, although the conservative treatment study should be applauded for showing reduced inpatient hospital stay, we would suggest that symptomatic patients who choose or are chosen by the treating physicians for intervention should be considered for the ambulatory treatment strategy, which is shown here to be effective and reasonably safe.

There are limitations to this study. Some eligible patients were not enrolled because they presented out of the 0900–1700 National Health Service working hours or when no trained members of staff were available. This factor introduces a potential recruitment bias, because patients presenting out of hours could be more unwell, and thus ambulatory management should be used cautiously in those patients. However, this prospective trial was pragmatic in its design and the patients who enrolled were likely to represent those suitable for ambulatory management. The study was necessarily open label with both patients and physicians aware of treatment allocation; however, the use of objective and identical discharge criteria were used for the primary outcome measure to ensure balance between treatment groups. Finally, not all patients in the standard care arm had pleural aspiration as initial management, which could have introduced bias towards the ambulatory arm. However, a post-hoc analysis excluding these patients showed that the primary outcome remained significantly different between the two arms.

Considering the data in the present study, what is now the optimal treatment for patients with primary spontaneous pneumothorax who require treatment? To our knowledge, our results show for the first time evidence of effective use of an entirely ambulatory strategy in patients with symptomatic primary spontaneous pneumothorax, as compared with current evidence based standard care. The ambulatory strategy was associated with reduced hospital stay and did not result in higher surgical or recurrence rates, but it did show an increase in adverse events related to readmission. Despite these readmissions, the total number of pleural procedures required and median hospital stay were significantly lower in patients in the ambulatory arm. These results have important implications for practice and suggest that future guidelines should include an ambulatory treatment option. We argue that this study provides strong evidence that the ambulatory strategy should replace the initial treatment (both aspiration and chest tube insertion) that forms the basis of current management guidelines. The ambulatory device used in this trial was the Pleural Vent (Rocket Medical). Ambulatory treatment might be just as effective with any ambulatory treatment strategy (eg, a chest drain attached to a one-way valve), but other devices were not tested in this study. It should be noted, however, that an ambulatory treatment strategy for primary spontaneous pneumothorax will require health-care services to develop ambulatory care facilities to allow patients to be safely followed up with the device in situ.

In conclusion, in patients with primary spontaneous pneumothorax, ambulatory management significantly reduces the duration of hospital stay during 30 days. Outpatient management is now a reasonable and probably preferable option in the management of this condition.

## Data sharing

All data requests should be submitted to the corresponding author (RJH) for consideration. Access to anonymised data may be granted for non-commercial research at the discretion of the Chief Investigator (NMR).
