# Supplementary material for: Ambulatory management of primary spontaneous pneumothorax: an open-label, randomised controlled trial
Source: Lancet. 2020 Jul 4;396(10243):39–49. doi: 10.1016/S0140-6736(20)31043-6 (PMC7607300; doi:10.1016/S0140-6736(20)31043-6)
Supplement: Supplementary appendix [file mmc1.pdf]

THE LANCET

Supplementary appendix

This appendix formed part of the original submission and has been peer reviewed. We post it as supplied by the authors.

Supplement to: Hallifax RJ, McKeown E, Sivakumar P, et al. Ambulatory management of primary spontaneous pneumothorax: an open-label, randomised controlled trial. *Lancet* 2020; **396:** 39–49.

Ambulatory management of primary spontaneous pneumothorax: an open-label randomised controlled trial

APPENDIX

CONTENTS:

Supplementary Figures & Table

- **Figure S1 –** Pleural Vent device (Rocket Medical, UK)
- **Figure S2 –** Histogram of total hospital stay by treatment arm
- **Figure S3 –** Kaplan-Meier survival plot of recurrence-free survival
- **Table S1** – Analgesia usage by treatment arm: Days 0 to 4. Proportion of patients requiring any analgesia and detail by analgesia type.
- **Table S2 –** Pneumothorax recurrence data up to 12 months: Total number of recurrences by treatment arm.
- **Table S3 –** Time to event analysis for first recurrence of pneumothorax up to 12 months, by treatment arm.

Study Protocol (V8.0)

Figure S1 – Pleural Vent device (Rocket Medical, UK)

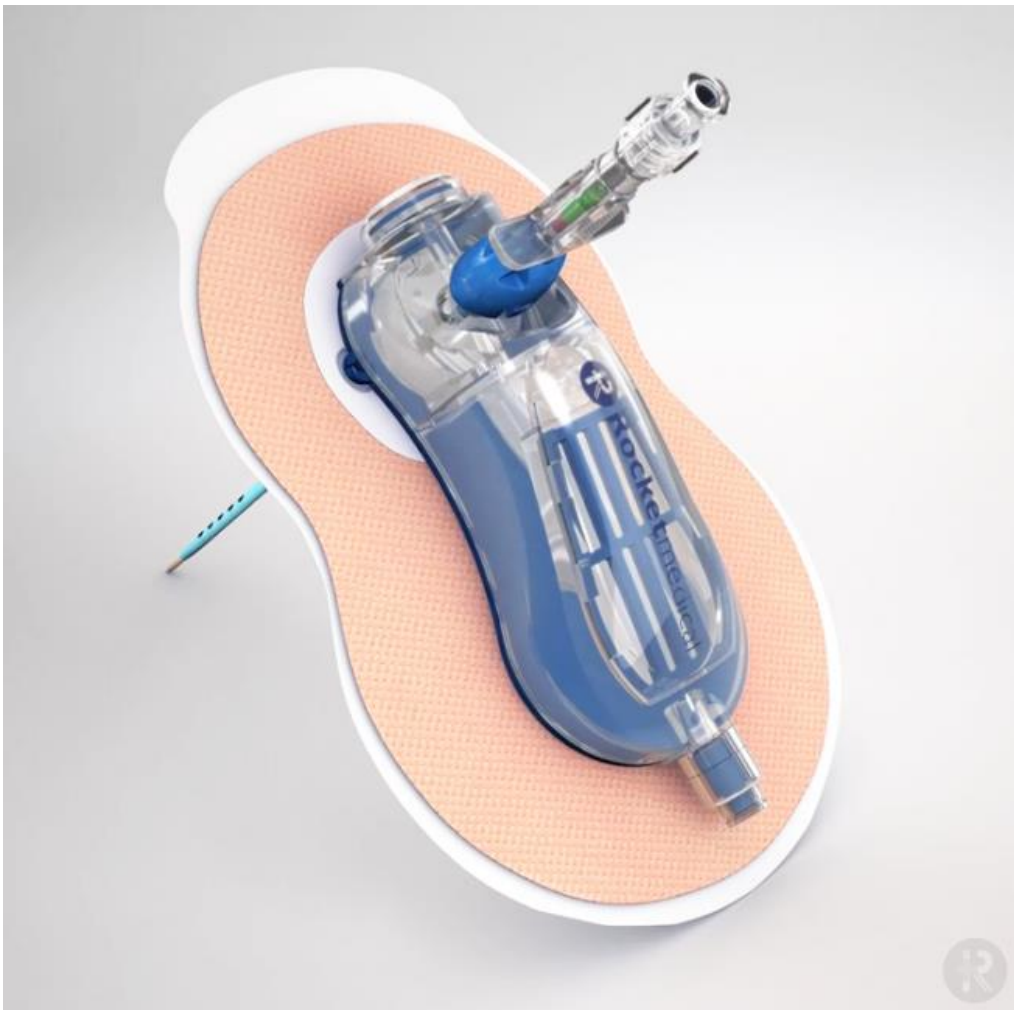

Figure S2 – Histogram of total hospital stay by treatment arm

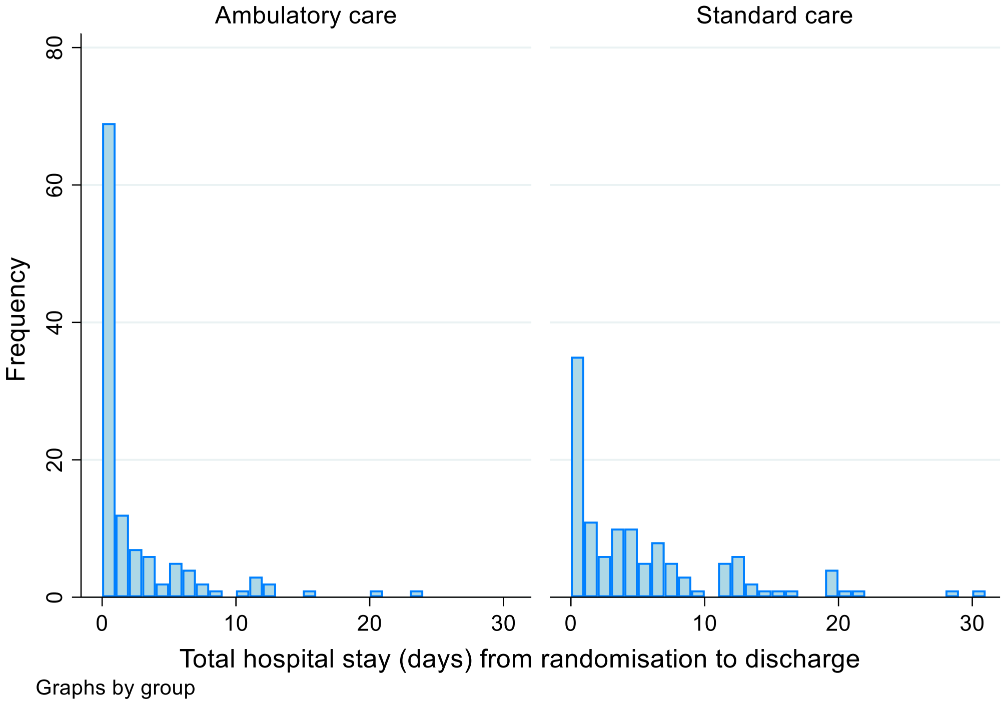

Figure S3 – Kaplan-Meier survival plot of recurrence-free survival

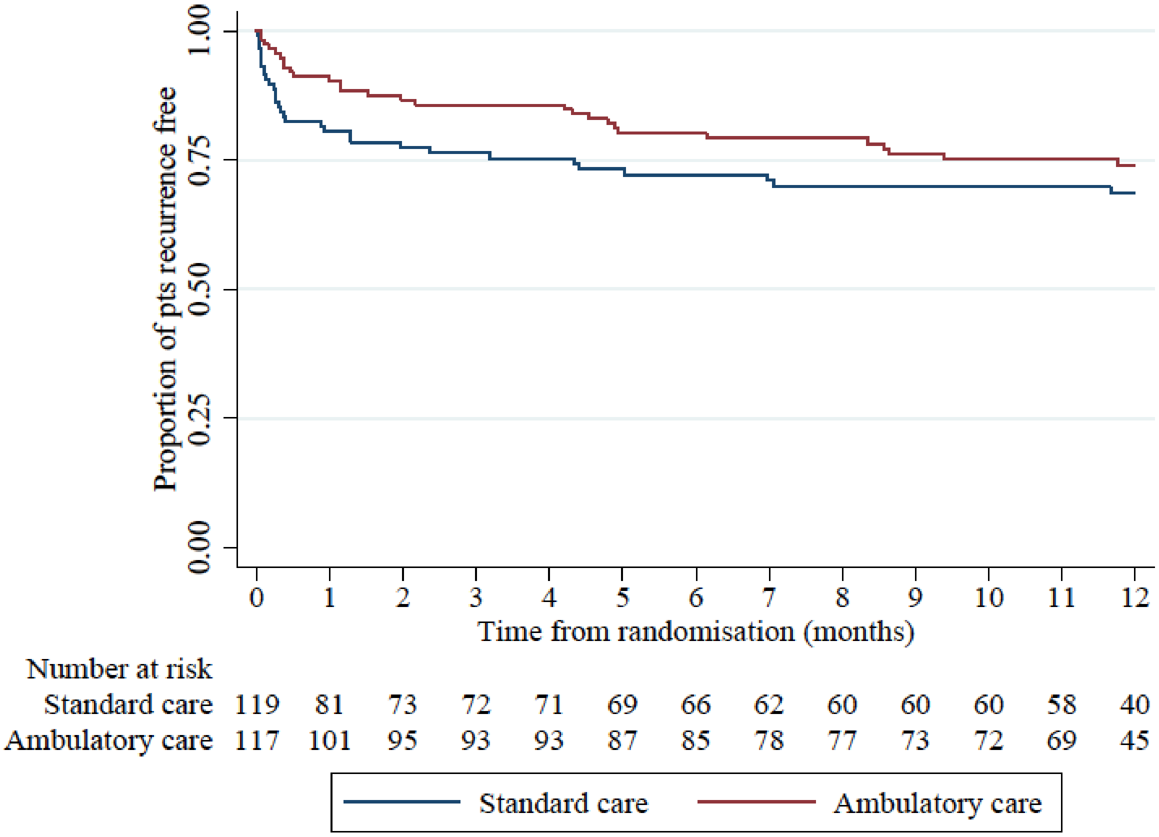

Table S1 – Analgesia usage by treatment arm: Days 0 to 4. Proportion of patients requiring any analgesia and detail by analgesia type.

	Ambulatory arm	Control arm	P value
**Patients requiring any analgesia on Day 0***	67.3% (74/110)	60.5% (69/114)	0.8856
**Paracetamol**			
Regular	29	33	
PRN	38	29	
**NSAIDS**			
Regular	17	14	
PRN	21	12	
**Codeine/Tramadol**			
Regular	10	18	
PRN	26	21	
**Opiates**			
Regular	0	1	
PRN	26	30	
**Patients requiring any analgesia on Day 1***	86.3% (88/102)	75.5% (71/94)	0.0549
**Paracetamol**			
Regular	40	44	
PRN	32	24	
**NSAIDS**			
Regular	23	20	
PRN	23	7	
**Codeine/Tramadol**			
Regular	22	19	
PRN	20	22	
**Opiates**			
Regular	1	2	
PRN	16	34	
**Patients requiring any analgesia on Day 2***	72.8% (59/81)	78.8% (52/66)	0.4042
**Paracetamol**			
Regular	35	39	
PRN	17	10	
**NSAIDS**			
Regular	23	23	
PRN	12	6	

**Codeine/Tramadol**				
	Regular	19	19	
	PRN	11	15	
**Opiates**				
	Regular	1	5	
	PRN	8	19	
**Patients requiring any analgesia on Day 3***		67.2% (43/64)	78.0% (39/50)	0.2023
**Paracetamol**				
	Regular	27	30	
	PRN	11	6	
**NSAIDS**				
	Regular	15	14	
	PRN	11	5	
**Codeine/Tramadol**				
	Regular	15	15	
	PRN	5	9	
**Opiates**				
	Regular	1	4	
	PRN	8	15	
**Patients requiring any analgesia on Day 4***		63.4% (26/41)	60.0% (21/35)	0.7600
**Paracetamol**				
	Regular	15	16	
	PRN	8	3	
**NSAIDS**				
	Regular	8	6	
	PRN	6	5	
**Codeine/Tramadol**				
	Regular	9	9	
	PRN	2	4	
**Opiates**				
	Regular	1	0	
	PRN	2	6	

*Data presented as number requiring analgesia/total number with data available. Data was collected up to completion of treatment, hence total number reduces each day

Table S2 – Pneumothorax recurrence data up to 12 months: Total number of recurrences by treatment arm.

	**Ambulatory Care n = 117**		**Standard Care n = 119**		**Difference (p value, where appropriate)**
**Within 30 days (n, %)**					
Ipsilateral					
• **Within 7days**					
○ **Ongoing (n, %)**	105	10 (10%)	107	16 (15%)	0.153
○ **New recurrence (n, %)**	105	8 (8%)	107	19 (18%)	0.022
• **7 – 30 days (n, %)**	100	5 (5%)	102	8 (8%)	0.678
Contralateral	100	0	102	0	
**1 - 6 months**					
• Ipsilateral	101	11 (11%)	92	13 (14%)	0.520
• Contralateral	101	11 (11%)	92	11 (12)	
		0		2 (2%)	
**6 - 12 months**					
• Ipsilateral	95	5 (5%)	86	3 (3%)	0.502
• Contralateral	95	5 (5%)	86	2 (2%)	
		0 (0%)		1 (1%)	

Table S3 – Time to event analysis for first recurrence of pneumothorax up to 12 months, by treatment arm.

	Ambulatory Care n = 117		Standard Care n = 119		HR (95% Confidence Interval)	(p value, from Cox model)
**Any recurrence within 12 months**	117	28 (24%)	119	36 (30%)	0.65 (0.40, 1.07)	0.090
**Ipsilateral recurrence within 12 months**	117	28 (24%)	119	33 (28%)	0.73 (0.44, 1.20)	0.215

Study Title: Randomised Ambulatory Management of Primary Pneumothorax (RAMPP)

OCTRU Trial No: CTU0018 **ISRCTN79151659** **Short title:** RAMPP

Ethics Ref: 15/SC/0240

Date and Version No: 8.0_03Nov2017

Chief Investigator: Prof Najib M. Rahman, *D Phil MSc MRCP*
Associate Professor of Respiratory Medicine, Oxford Centre for Respiratory Medicine
Clinical Director, Oxford Respiratory Trials Unit
Churchill Hospital,
Oxford, UK OX3 7LE
Email: najib.rahman@ndm.ox.ac.uk

Trial Clinical Co-ordinator Dr Rob Hallifax
Clinical Research Fellow, Oxford Respiratory Trials Unit
OCTRU – Respiratory Division, University of Oxford
Oxford Centre for Respiratory Medicine
Churchill Hospital
Oxford, UK OX3 7LE
Email: Rob.Hallifax@ouh.nhs.uk

Sponsor: University of Oxford

Funder: NIHR Research For Patient Benefit (RfPB) grant and MRC Training Fellowship (For Dr R Hallifax)

Chief Investigator Signature:

There are no potential conflicts of interest.

Confidentiality Statement

This document contains confidential information that must not be disclosed to anyone other than the Sponsor, the Investigator Team, host organisation, and members of the Research Ethics Committee, unless authorised to do so.

© Copyright: The University of Oxford and Oxford University Hospitals NHS Trust 2014

TABLE OF CONTENTS

1. SYNOPSIS 12
2. ABBREVIATIONS 14
3. BACKGROUND AND RATIONALE 15
4. OBJECTIVES AND OUTCOME MEASURES 18
5. STUDY DESIGN 20
6. PARTICIPANT IDENTIFICATION 20
6.1. Study Participants 20
6.2. Inclusion Criteria 20
6.3. Exclusion Criteria 20
7. STUDY PROCEDURES 21
7.1. Recruitment 21
7.2. Informed Consent 21
7.3. Screening and Eligibility Assessment 22
7.4. Co-enrolment Guidelines 22
7.5. Randomisation, blinding and code-breaking 22
7.6. Study Assessments 23
7.7. Follow-up Visits (All patients) 25
8. INTERVENTIONS 27
9. SAFETY REPORTING 30
9.1. Definition of Adverse Event (AE) and Serious Adverse Event (SAE) 30
9.2. Expected Adverse Events 31
9.3. Recording and Reporting Procedures 32
9.3.4. Following reporting 33
9.4. Ambulatory Device (Rocket Pleural Vent) Safety Testing 33
10. STATISTICS AND ANALYSIS 33
10.1. Number of Participants 33
10.2. Analysis of Endpoints 34
10.3. Interim Monitoring and Analyses 36
10.4. Analysis Plan (Summary) 36
10.5. Measures of compliance and adherence 36
11. DATA MANAGEMENT 36
11.1. Access to Data 36
11.2. Data Recording and Record Keeping 36

© Copyright: The University of Oxford and Oxford University Hospitals NHS Trust 2014

12. QUALITY ASSURANCE PROCEDURES 38
12.1. Study infrastructure 38
12.1.1. Trial Management Group (TMG) 38
12.1.2. Trial Steering Committee (TSC) 38
12.1.3. Data Safety Monitoring Committee (DSMC) 38
13. ETHICAL AND REGULATORY CONSIDERATIONS 39
13.1. Declaration of Helsinki 39
13.2. Guidelines for Good Clinical Practice 39
13.3. Approvals 39
13.4. Reporting 39
13.5. Participant Confidentiality 39
13.6. Expenses and Benefits 40
13.7. Other Ethical Considerations 40
14. FINANCE AND INSURANCE 40
14.1. Funding 40
14.2. Insurance 40
15. PUBLICATION POLICY 40
16. REFERENCES 42
17. APPENDIX A: STUDY FLOW CHART 44
* "Sufficient re-expansion" is defined as complete or almost complete re-expansion (only a very small (<1cm) rim of air apically) on CXR. 44
** On going air leak assessed by attempted aspiration through the device using a syringe and connector: if device patent (moving in-built diaphragm) but unable to aspirate then there is no ongoing air leak; if able to aspirate air freely, then there is ongoing leak and active pneumothorax. 44
18. APPENDIX B: SCHEDULE OF STUDY PROCEDURES 45
19. APPENDIX C: AMENDMENT HISTORY 46

© Copyright: The University of Oxford and Oxford University Hospitals NHS Trust 2014

1. SYNOPSIS

**Study Title**	Randomised Ambulatory Management of Primary Pneumothorax (RAMPP)	
**Internal ref. no. / short title**	RAMPP	
**Study Design**	Multi-centre randomised controlled trial comparing ambulatory management to standard care (aspiration +/- standard chest drain insertion) with an observational cohort study of patients not requiring an intervention.	
**Study Participants**	Patients with primary spontaneous pneumothorax (PSP)	
**Planned Sample Size**	236 in intervention and control arm; and concurrent observational cohort study (no upper limit)	
**Planned Study Period**	19May2015 – 31Dec2018	
	**Objectives**	**Outcome Measures**
**Primary**	Assess whether use of an ambulatory device (Rocket Pleural Vent) and treatment strategy reduces hospital stay.	Total length of stay in hospital (including re-admissions) up to 30 days post randomisation.
**Secondary**	1. Determine whether digitally measured air leak (using Thopaz device) and its evolution over treatment, can predict short term clinical trajectory in patients with pneumothorax, including requirement for prolonged drainage and need for thoracic surgical intervention.	a) Digitally measured air leak: days 0 -4 (or until chest drain/device removal)<br>b) Rate of surgical referral/failure of medical therapy (at day 4).<br>c) Number of pleural procedures required during primary admission.<br>d) Rate of lung re-expansion by comparing the percentage of the hemithorax occupied by the pneumothorax using daily Chest Radiograph (CXR scoring system).
	2. Establish whether radiological evidence (on CT scanning) of emphysema-like changes (ELC) and inflammation can predict long term outcome (i.e. recurrence rate at 12 months follow-up).	Recurrence rate at 1 week post **completion of treatment*** and 1, 6 and 12 months post enrolment assessed at follow-up clinic.<br>Serum highly sensitive C-reactive protein (CRP) level on blood test (at baseline).
	3. Assess whether ambulatory care and early discharge is safe and cost-effective in the treatment of PSP.	a) Rate of complications: intervention site bleeding or infection, blockage of ambulatory device (Rocket Pleural Vent) and need for additional procedure.<br>b) NHS-related healthcare costs by including use of equipment and devices, consumables, medications, and staff and theatre, initial and subsequent hospitalisations over the 12

© Copyright: The University of Oxford and Oxford University Hospitals NHS Trust 2014

		months follow-up, and outpatient contacts.<br>c) Incremental cost per QALY gained when ambulatory care is compared with standard care.
	4. Determine whether patient experience is improved with an ambulatory device (Rocket Pleural Vent): pain of procedure, breathlessness, quality of life assessments (EQ-5D-5L), and time to return to working status.	a) Patient related factors: procedural pain/discomfort and breathlessness on VAS scoring system, analgesia usage, and generic health-related and disease-specific quality of life measures.<br>b) Time to return to work and total days off work.
	5. Assess recurrence rate of pneumothorax	Radiological evidence (CXR or Computed Tomography (CT) scan) of recurrence 1 week post completion of treatment and 1, 6 and 12 months post enrolment.
**Intervention**	If a pleural procedure is required (i.e. large/symptomatic pneumothorax) the patient will be randomised 1:1 to either:<br>1) "Intervention" arm: ambulatory device (Rocket Pleural Vent) inserted.<br>2) "Control" arm: aspiration +/- standard chest drain insertion with underwater seal (as per BTS guidelines).<br><br>Patients who do not require a pleural procedure (if pneumothorax is small/asymptomatic) will be invited to participate in an observational cohort study.	

***Completion of treatment** is defined as a successful aspiration or chest drain/device removal for the randomised controlled trial. In the observational cohort study, completion of treatment is defined as discharge home post initial hospital assessment.

© Copyright: The University of Oxford and Oxford University Hospitals NHS Trust 2014

2. ABBREVIATIONS

A&E	Accident & Emergency Department
AE	Adverse Event
CI	Chief Investigator
CRF	Case Report Form
CT	Computerised Tomography
CTRG	Clinical Trials & Research Governance, University of Oxford, UK
CXR	Chest Radiograph (X-ray)
EQ-5D-5L	Euroqol (5 dimensions, 5 level): generic health-related quality of life measure
ELC	Emphysema-like Changes
GCP	Good Clinical Practice
HRCT	High Resolution Computed Tomography
ICER	Incremental Cost-Effectiveness Ratio
ICH	International Conference on Harmonisation of Technical Requirements for Registration of Pharmaceuticals for Human Use
DSMC	Data Safety Monitoring Committee
MAU	Medical Assessment Unit
MSD IT	Medical Sciences Division IT
NHS	National Health Service, UK
OCTRU	Oxford Clinical Trials Research Unit
ORTU	Oxford Respiratory Trials Unit, UK
PAL	Prolonged Air Leak
PI	Principal Investigator
PIL	Participant/ Patient Information Leaflet
PSP	Primary spontaneous pneumothorax
QALY	Quality Adjusted Life Expectancy
QoL	Quality of Life
R&D	Research &Development Department (NHS Trust)
RAMPP	Randomised Ambulatory Management of Primary Pneumothorax (Study title)
REC	Research Ethics Committee
SAE	Serious Adverse Event
SOP	Standard Operating Procedure
TMG	Trial Management Group
TSC	Trial Steering Committee
TSI	Trial Specific Instructions

© Copyright: The University of Oxford and Oxford University Hospitals NHS Trust 2014

UK	United Kingdom
VAS	Visual Analogue Scale
VATS	Video-Assisted Thoracoscopic Surgery

3. BACKGROUND AND RATIONALE

i. Importance

Pneumothorax - air in the pleural space - is a common pathology. Primary spontaneous pneumothorax (PSP) conventionally refers to patients developing a pneumothorax, in the absence of trauma, with no underlying established lung pathology. PSP occurs in ~3,000 patients per year in the UK [1]. A minority can be managed conservatively with close observation only. However, most patients will require an intervention to re-expand the lung. In most patients "aspiration" of the trapped air using a cannula and syringe is considered, but more than 50% will require treatment with insertion of a chest drain and a standard underwater seal. The average duration of in-patient stay of patients admitted for drainage is 6-8 days [2]. Patients who do not resolve their pneumothorax during this drainage period, with “prolonged air leak” (PAL), require in-patient referral to a thoracic surgical team for consideration for further intervention. However, the evidence base for treatment in this condition is poor - the length of in-patient drainage required, prediction of PAL and need for inpatient surgical correction and recurrence after first episode of pneumothorax is, at present, unpredictable, leading to the non-selected treatment of all patients with a period of chest drainage, resulting in prolonged hospital stay. The decision to proceed to surgical management for PAL is taken after this period of uncertain observation, and there is no robust evidence informing the optimal timing of surgical referral. This non-specific, generic treatment is likely to cause significant delays in referral and prolonged hospital stays. Longer hospital admissions increase the risk of venous thromboembolism from lack of mobility and hospital acquired infection. Patients with PSP tend to be young who would otherwise return to work sooner. Combining data from 11 studies (from 1963-1995), the recurrence rate for PSP is 30%, with a range of 16-52 % [3]. No subsequent studies have been able to more precisely define recurrence rate, or more importantly, which patients are most at risk. At present, UK guidelines do not advocate surgical intervention after a single episode of PSP [4].

ii. Pathophysiology

PSP is more common in taller patients with low body-mass index (BMI) and smokers. Although PSP occurs in patients without evidence of underlying established lung pathology, most patients have emphysema-like changes (ELC), i.e. blebs and bullae, in the lungs on Computed Tomography (CT) scans. One study found these changes bilaterally in the upper lung zones in 81% of non-smoking patients with PSP but not in healthy controls [5]. Thoracoscopic investigation of 250 healthy individuals with no prior history of pleural disease (treated with thoracoscopic sympathectomy) demonstrated a 6% incidence of apical blebs. These were more prevalent in slim individuals (BMI <22kg/m^2) who smoked [6].

However, it is unclear how often these lesions are the actual site of air leakage. A landmark paper has examined patients with PSP at thoracoscopy using inhaled fluorescein-enhanced autofluorescence has demonstrated air leaks in PSP in areas of parenchymal abnormality or

© Copyright: The University of Oxford and Oxford University Hospitals NHS Trust 2014

"pleural porosity". These areas appear normal on plain white light thoracoscopy, and do not necessarily correspond to areas with blebs and bullae [7]. These areas of pleural porosity are described as areas of disrupted mesothelial cells at the visceral pleura replaced by an inflammatory elastofibrotic layer with increased porosity, allowing air leakage. Lower lung density measurements on CT scan in patients with PSP (compared to controls) support a hypothesis that airways inflammation may lead to obstruction and air-trapping within the peripheral lung parenchyma giving rise to the observed porosity [8]. This corroborates older pathological studies of lung, surgically-resected to treat PSP recurrence, which found fibrosis and chronic inflammation [9]. Although many factors have been implicated, the exact pathogenesis of PSP, and how that correlates with clinically important outcomes such as length of air leak, requirement for prolonged drainage and need for preventive surgical correction, remains unknown.

iii. Pilot Data on Air Leak

When standard management does not sufficiently resolve the air leak (resulting in PAL), surgical referral is recommended [4]. However, the optimal timing of surgical intervention is unknown. Current guidelines suggest that in-patients with a persistent air leak or failure of the lung to re-expand an early (3-5 days) thoracic surgical opinion should be sought. However, there is very little evidence underpinning this practice and there are no published data on prediction of PAL or need for in-patient surgical intervention in pneumothorax. Management is thus not tailored and generic in all cases, leading to prolonged and potentially unnecessary hospital stay. To begin to address this issue, we have assessed 9 patients with pneumothorax using a recently available digital suction device (Thopaz), which is able to accurately measure the degree of air leak during drainage. Although the treating clinical teams were blind to air leak measurement results, there was a large difference in mean early leak measurement (504ml/min vs 77ml/min) between those patients who required surgery and those spontaneously resolving, although this did not reach statistical significance subject to sample size limitation. However, our use of the digital suction device post-thoracoscopy (during which a pneumothorax is induced) has demonstrated that initial measurements have the potential to predict which patients are likely to have non-expandable or trapped lung (data in manuscript phase). These pilot studies suggest that accurate digital measurement of initial air leak post-drain insertion is likely to be a powerful surrogate marker for PAL, the potential for lung expansion and hence non-resolving pneumothorax, providing the testable hypothesis base for this application. This novel measurement has not before been assessed in the clinical management of pneumothorax and has potentially a large clinical impact.

iv. Ambulatory Management of Pneumothorax

Reducing the need for chest drains with bulky underwater systems may allow patients to be more mobile and facilitate earlier discharge. A "Heimlich valve" (one-way valve connected to a chest drain, rather than a bulky underwater seal) has been previously proposed, in the form of either one-way valves attached to standard chest drains or the relatively new "pocket" devices, in which the drainage catheter and one way valve are integrated in to a single device.

A number of small studies show feasibility of outpatient management for PSP. A case series of 226 patients with PSP managed by observation or flutter valve concluded that outpatient management was "safe, efficient, and economical" [10]. A randomised trial of 30 PSP patients (17 given "thoracic vent", 13 given standard chest drain) showed no significant difference in complications

© Copyright: The University of Oxford and Oxford University Hospitals NHS Trust 2014

or re-expansion rates, but 70% of "vent" patients were managed as outpatients and required fewer analgesics, with patients in the control group (standard chest drain) hospitalised for 8 days [2]. An observational study using the same device in 35 patients concluded 89% could be managed as outpatients, and the device was significantly less painful than standard treatment [11]. These findings suggest that ambulatory and out-patient management is feasible, but more definitive data comparing this treatment to standard care is now required before widespread uptake of this treatment option occurs in clinical practice.

Other studies have assessed efficacy of the one-way Heimlich valve attached to a standard chest drain in PSP, but there are no randomised controlled trials and 2 case-controlled studies showing differing results. The first compared 47 patients with PSP with standard chest drain to 20 managed with Heimlich ambulatory drain and demonstrated similar rates of re-expansion (70-75%) and requirement for surgery [12]. The second study compared 47 patients with PSP treated with small-bore chest drain and a Heimlich valve to 47 patients treated with chest drainage. The Heimlich valve group achieved a lower success rate (47% vs 89%) [13]. A retrospective case series of 240 patients (PSP and air leak post-surgical resection) who were discharged with a drain and Heimlich valve demonstrated a 4% "failure rate" requiring hospital readmission [14]. Another five non-randomised, observational or retrospective studies (totalling 195 patients) also describe high rates of success with outpatient management of PSP with one-way valves [15] [16] [17] [18] [19]. These data are well summarised in a 2013 systematic review of 18 studies using ambulatory management with Heimlich valve, reporting an overall success rate of 85.8% and successful outpatient management in 77.9% with "few complications". However, the evidence was of poor quality with a high risk of bias, consisting mainly of only two small randomised trials with the remainder being case series [20].

For the purpose of this trial we will use a Rocket Pleural Vent supplied by Rocket Medical as an ambulatory device. This device has been designed for treatment of spontaneous, iatrogenic or traumatic simple pneumothorax.

v. Surgical Treatment Options and Lessons from Ambulation post-Surgery

When continued air leak occurs during drainage of PSP (traditionally after 7 days of drainage in hospital), surgical referral is recommended to repair the air leak [4]. The majority of PSP air leaks will spontaneously resolve if the pneumothorax size is controlled with drainage over a period of 7-10 days, and hence the optimal timing of surgical intervention is unknown. Early surgical intervention may prevent prolonged hospital stay, but may result in a large number of patients undergoing invasive thoracic surgery (with its attendant long term potential complications) unnecessarily.

vi. Need for the trial

Better predictive data on which patients will have PAL at baseline and which will need further intervention or suffer recurrence has the important potential to allow the selective and targeted treatment of patients according to likely outcome, resulting in more personalised treatment. The proposed study aims to provide a robust evaluation of ambulatory strategy against the standard

© Copyright: The University of Oxford and Oxford University Hospitals NHS Trust 2014

management guidelines, and assess clinically important outcomes to definitively answer whether ambulatory treatment has advantages and should therefore be taken up in to standard clinical practice.

4. OBJECTIVES AND OUTCOME MEASURES

Objectives	Outcome Measures	Timepoint(s) of evaluation of this outcome measure (if applicable)
**Primary Objective**<br>To assess whether use of an ambulatory device (Rocket Pleural Vent) and treatment strategy reduces hospital stay.	Total length of stay in hospital up to 30 days post randomisation.	Up to 30 days post randomisation.
**Secondary Objectives**<br><br>1. Determine whether digitally measured air leak and its evolution over treatment, can predict short term clinical trajectory in patients with pneumothorax, including requirement for prolonged drainage and need for thoracic surgical intervention.	Digitally measured air leak (using Thopaz device).	From day 0 (immediately post intervention) to day 4 (or until the chest drain/device removal).
	Rate of surgical referral/failure of medical therapy.<br><br>*Note: For the purposes of analysis and outcome, "referral" to thoracic surgery is used as the outcome rather than actual occurrence of surgical intervention, due to variable delays in the provision of surgical beds in different centres. Referral for surgery is thus a measure of "failure" of medical treatment (i.e. intercostal drain insertion).*<br><br>*Note: Data on digital air leak will not be available to clinicians to guide surgical referral decision-making in order to objectively test its validity, and the objective criteria above will be recorded on the E-CRFs.*	On day 4.
	Number of pleural procedures required during primary admission.	From primary admission until completion of treatment.
	Rate of lung re-expansion by comparing the percentage of the hemithorax occupied by the pneumothorax using a CXR scoring system.	Daily until completion of treatment.

© Copyright: The University of Oxford and Oxford University Hospitals NHS Trust 2014

2. Establish whether radiological evidence (on CT scanning) of emphysema-like changes (ELC) and inflammation, and serum markers of inflammation can predict long term outcome.	CT evidence of ELC and inflammation: number and size of bullae at apices, and bronchial wall thickness in lung apices (surrogate for inflammation).<br><br>Correlation with recurrence rate (and time to recurrence) assessed at follow-up clinic.<br><br>Serum highly sensitive C-reactive protein (CRP) level on blood test.	At 1 week post completion of treatment and 1, 6 and 12 months post enrolment assessed at follow-up clinic.<br><br>Baseline.
3. Assess whether ambulatory care and early discharge is safe and cost-effective in the treatment of PSP.	Rate of complications: intervention site bleeding or infection, blockage of device and need for additional procedure.	From initial admission until 1 week post completion of treatment.
	NHS-related healthcare costs by including use of equipment and devices, consumables, medications, and staff and theatre, initial and subsequent hospitalisations over the 12 month follow-up, and outpatient contacts.<br><br>Incremental cost per QALY gained when ambulatory care is compared to standard care.	From initial admission throughout follow-up.
4. Determine whether patient experience is improved with an ambulatory device (Rocket Pleural Vent): pain of procedure, breathlessness, quality of life assessments (EQ-5D-5L), and time to return to working status.	Patient related factors: procedural pain/discomfort and breathlessness on VAS scoring system, analgesia usage, and generic health-related and disease-specific quality of life as measured using the Euroqol 5 dimensions 5 levels (EQ-5D-5L).	From initial admission throughout follow-up.
	Time to return to work and total days off work.	From initial admission throughout follow-up.
5. Assess recurrence rate of pneumothorax.	Radiological evidence (CXR and or CT) of recurrence.	At 1 week post completion of treatment and at 1, 6 and 12 months post enrolment assessed at follow-up clinic.

© Copyright: The University of Oxford and Oxford University Hospitals NHS Trust 2014

5. STUDY DESIGN

RAMPP is a multi-centre interventional randomised controlled trial comparing ambulatory to standard management of primary pneumothorax in patients requiring an intervention. Patients who do not require an intervention and/or are asymptomatic will be invited to participate in an observational cohort study.

6. PARTICIPANT IDENTIFICATION

6.1. Study Participants

All patients presenting with spontaneous pneumothorax to Accident & Emergency (A&E) or the acute medical teams will be assessed for eligibility:

- Those patients who do not require intervention (according to national British Thoracic Society (BTS) evidence-based guidelines) will be invited to participate in an observational cohort study. Baseline data and follow-up/outcome data will be collected to provide epidemiological data on recurrence rates.
- Those patients who do require intervention will be invited to give consent to be randomised to one of the two interventional arms of the trial.

6.2. Inclusion Criteria

1. Presenting with primary spontaneous pneumothorax as confirmed by a chest radiograph or a CT scan.
2. Age >= 16* years old and <= 55 years old.
3. Ability to consent to participation.

**Common law presumes that young people aged between 16 and 18 are usually competent to give consent to treatment and consent from those with parental responsibility is not legally necessary. Eligible young persons believed to be competent by the PI or delegate should be approached about the study. The involvement of parents in decision-making should be encouraged unless the young person objects.*

6.3. Exclusion Criteria

1. Known or suspected underlying lung disease (including >20 pack year smoking history).**
2. Evidence of tension pneumothorax (these patients should be treated immediately as medical emergencies).
3. Females who are pregnant or lactating.
4. Inability to consent or comply with the trial requirements.
5. Contraindication to thoracic procedure. (Only applies to patients being enrolled into Intervention or Control arms – i.e. not observational cohort).
6. Any other significant disease or disorder which, in the opinion of the Investigator, may either put the participants at risk because of participation in the trial, or may influence the result of the trial, or the participant's ability to participate in the trial.

** "Childhood asthma" is not considered an exclusion criterion. Patients with a diagnosis of asthma in childhood/young adulthood who do not require the use of a regular "preventer" inhaler (i.e. inhaler

© Copyright: The University of Oxford and Oxford University Hospitals NHS Trust 2014

containing a steroid or long-acting beta-agonist), and only occasionally use a "reliever" inhaler (short-acting beta-agonist) and have never been hospitalised due to asthma remain eligible for participation in this study.

7. STUDY PROCEDURES

Refer to Appendix B for a schedule of study procedures.

7.1. Recruitment

RAMPP is a multi-centre trial involving centres across the UK with specific interest and experience in recruiting to large pleural studies. The co-ordinating centre will be the Oxford Respiratory Trials Unit ORTU), a therapeutic sub-division of the Oxford Clinical Trials Research Unit (OCTRU), University of Oxford) based at the Churchill Hospital, Oxford.

Participants will be screened from normal clinical care, which for patients presenting with pneumothorax will usually occur in the Accident and Emergency (A&E) departments or as a direct referral to the general medical on-call team. As such, screening will occur early in the patient's treatment pathway. Those identified as being eligible for enrolment will be approached by the clinician responsible for their care and provided with written information about the study. In recruiting centres, whose respiratory team are in close proximity to A&E, the respiratory team may be involved from the outset, otherwise initial screening, consent and enrolment will occur by the A&E or general medical on-call teams. These teams will be specifically trained by the Trial Clinical Co-ordinator or the local Principal Investigator (PI).

Patients requiring intervention can be enrolled and randomised up to 24hours after presentation (as long as they still have an ongoing symptomatic pneumothorax, despite initial intervention). See Section 8 "Intervention".

Patients not requiring intervention are eligible for enrolment into the Observational Cohort. These patients can be enrolled after they have been discharged from A&E (up to 2 weeks). The patient can be contacted by the responsible clinician to be made aware of the trial (by either phone or post).

Responsibility for patient enrolment will lie with the local investigator, who should be contacted by the responsible clinician to agree inclusion criteria are met if possible.

Recruiting centres will keep records of all patients screened and this data will be regularly transferred to Oxford Respiratory Trials Unit (ORTU) for centralised review and record keeping.

7.2. Informed Consent

Once an eligible patient is identified and agrees to participate in the study, informed written consent will be obtained by the principal investigator or other suitably qualified delegated personnel. The participant will be asked to sign and date the latest approved version of the Informed Consent form before any study specific procedures are performed.

Written and verbal versions of the Participant Information and Informed Consent will be presented to the participants detailing no less than: the exact nature of the study; what it will involve for the participant; the implications and constraints of the protocol; the known side effects and any risks involved in taking part. It will be clearly stated that the participant is free to withdraw from the study at

© Copyright: The University of Oxford and Oxford University Hospitals NHS Trust 2014

any time for any reason without prejudice to future care, and with no obligation to give the reason for withdrawal.

As pneumothorax is an acute medical problem, it would not be appropriate to wait the usual 24 hours to allow patients time to read the patient information leaflets, prior to intervention. Therefore, ideally the patient should still be given a reasonable short period of time to read, digest and ask questions about the study, prior to an approach for consent. Written Informed Consent will then be obtained by means of participant dated signature and dated signature of the person who presented and obtained the Informed Consent. The person who obtained the consent must be suitably qualified and experienced, and have been authorised to do so by the local Principal Investigator. A copy of the signed Informed Consent form will be given to the participant. The original signed form will be retained at the study site, a copy will be filed with patient's medical records and another copy will also be transferred to the ORTU for the purpose of central monitoring and to confirm patient's consent to have a blood sample taken for storage at ORTU.

7.3. Screening and Eligibility Assessment

Patients with a pneumothorax will be identified by way of chest radiograph or (rarely) found on CT scan. If there is any evidence of tension pneumothorax (see exclusion criteria), the responsible team should treat the patient immediately. Patients may be enrolled up to 24 hours after presentation, i.e. even after initial intervention (see Appendix A).

The Clinical Trial Co-ordinator based at ORTU will be on call for advice and to assist with pleural interventions during office hours locally (Oxford). At trial set-up, training will be provided for research teams, senior A&E and Medical staff (e.g. in Medical Assessment Unit (MAU) at each site with regards to the trial protocol and familiarity with the ambulatory device (Rocket Pleural Vent) to allow recruitment to occur outside office hours.

7.4. Co-enrolment Guidelines

Once the patient is recruited into this study, they should not be concurrently enrolled into any other trial which requires pleural intervention. Patients already taking part in other trials requiring pleural intervention should not be enrolled into this study, however, a decision as to whether a patient may be entered into this study will be made on a case by case basis at the discretion of the Chief Investigator.

7.5. Randomisation, blinding and code-breaking

Once written consent is obtained and the patient has been randomised, the patient will be assigned a unique patient trial number that will then be used on the baseline e-CRF (including basic demographic data) and for all subsequent e-CRFs.

The randomisation process will use a centralised web-based randomisation system provided by the Oxford Clinical Trials Research Unit (OCTRU) which will also hold the randomisation list. Minimisation with a residual randomised component will occur, for the minimisation factors of centre and size of pneumothorax (≥4cm vs <4cm) at presentation.

Due to the nature of the interventions, patients and clinicians cannot be blinded to allocation and therefore code-breaking is not needed for this trial. However, the objective "fitness for discharge" data will be blind reviewed after the trial by an independent assessor blind to treatment arm (i.e. objective

© Copyright: The University of Oxford and Oxford University Hospitals NHS Trust 2014

blind outcome assessment) and compared to actual time spent in hospital at study end. Recurrence rates at 12 months will be blindly assessed by an independent assessor by reviewing patients CXR and hospital records for re-admissions at study end. In addition, the clinician responsible for making a decision to discharge a patient will be blinded to the air leak measurements recorded as part of the trial protocol.

7.6. Study Assessments

a. Collection of baseline clinical data (All patients)

This includes demographics, details of clinical history and past medical history, symptoms, drug history, smoking (tobacco and marijuana) history, morphometric data (height and weight) and baseline clinical observations (heart rate, respiratory rate, oxygen requirement, blood pressure), as recorded in normal clinical care.

b. Blood tests (All patients)

As part of routine clinical care, all patients will have baseline blood tests (haemoglobin, white cell count, platelet count, electrolytes, liver function tests and clotting tests). These results will be documented on the e-CRFs. Patients will be consented to have an additional blood test to measure inflammation (called highly sensitive C-reactive protein (CRP)). (This trial sample should ideally be taken at the same time as standard care bloods (on day 0 or 1)). These blood samples will be sent to the Oxford Respiratory Trials Unit for analysis and but will also be stored as part of the ORTU Collection of the Oxford Radcliffe Biobank and used in future studies (e.g. to determine genetic factors predisposing to pneumothorax).

c. Chest radiographs (CXR) (All patients)

All participants will undergo a number of plain chest radiographs, which should preferably be PA erect. Electronic copies of all chest radiographs should be uploaded directly onto the study database where possible, and if not sent via CD/disk to ORTU. The CXR should be coded with the trial number only to ensure patients' confidentiality is maintained.

d. Daily clinical data (Intervention and Control arm only)

In addition to a daily CXR, whilst an in-patient, all patients will have baseline clinical observations (heart rate, respiratory rate, oxygen requirement, blood pressure), recorded on the e-CRF. The responsible clinician should also check the chest drain site, looking for evidence of infection, haematoma or subcutaneous emphysema.

In the standard (chest drain) management arm, the drain should be assessed daily for bubbling (i.e. ongoing air leak and swinging (evidence of chest drain still being patent and in the pleural space).

e. Digital air leak measurement (Intervention and Control arm only)

All patients requiring pleural intervention will have digital measurement of air leak using a digital measuring device called Thopaz. Measurements will be taken immediately post-intervention (day 0) of ambulatory or chest drain, then daily at around the same time on days 1 to 4 (or until chest drain/device removal). On each occasion the Thopaz device should be attached to the chest drainage device (either ambulatory device or standard chest drain, dependent on the arm of the trial) for 10 minutes, with the device set in gravity mode (i.e. providing no suction, at -0.4kPa). During this time, the air leak measurement should be recorded manually by reading the measurement from the device at: 1 minute, 5

© Copyright: The University of Oxford and Oxford University Hospitals NHS Trust 2014

minutes, and finally 10 minutes. Average air leak measurement will be captured digitally on the suction device, and will be downloaded after discharge to be analysed anonymously at a later date. The downloaded data should be emailed to a dedicated RAMPP email address as per the Trial Specific Instructions (also detailing how the digital suction device and measurements should be performed and downloaded).

f. Questionnaires and VAS (Visual Analogue Scale) assessment

All patients will be asked to complete the EQ-5D-5L questionnaire (at baseline/on the day of admission and then at 1 week post completion of treatment and at 1, 6 and 12 month post enrolment) and the Visual Analogue Scale assessment to measure thoracic pain and breathlessness (at baseline, daily with device/drain in situ and at follow-up – as above). These will be completed by patients on paper CRFs and originals posted to ORTU for scoring and data entry.

g. Failure of Medical Treatment / Surgical Referrals (Intervention and Control arm only)

There is no robust evidence on the ideal timing for surgical intervention. Current BTS guidelines suggest that cases of persistent air leak or non-re-expansion should be referred after 3-5 days [4]. To achieve objective outcomes for this study, the following measureable and documentable criteria have been developed to ensure consistent practice, and will be recorded as part of the study in all cases. Referral for thoracic surgery will occur in the presence of ALL of the following:

1. Day 4 post-insertion of chest drain, persistent air leak as measured by "bubbling" chest drain attached to underwater seal, or evidence of on-going air leak through an ambulatory device.
2. Persistent pneumothorax on CXR.
3. Patient agreement.
4. No contraindication to thoracic surgery.

Patients referred for surgery will undergo Computed Tomography (CT) scan to detect lung parenchymal abnormalities prior to surgery, as is usual practice. All cases and radiology (both those referred to surgery and those successfully treated with medical management) will be blindly assessed at the end of the study to ensure the discharge and surgery criteria were robustly followed.

h. “Fitness for Discharge” assessment

The “fitness of discharge” criteria should be completed on all patients to allow comparison between control and interventional arms. They are intended as a guide for the responsible clinician, but the decision to discharge lies with their clinical judgement.

The criteria are:

- Patient agreement.
- Clinically stable cardio-respiratory observations (Oxygen saturation, respiratory and heart rate, blood pressure, i.e. Early Warning Score (EWS) of 0 or 1).
- No increase in size of pneumothorax on serial CXRs.
- Not requiring oxygen or other ventilator assistance.
- Patient mobile and independent to self-care.
- Written information on point of contact if concerns and follow-up plan.
- Patient lives with a responsible person at home and is able to help patient if required.

© Copyright: The University of Oxford and Oxford University Hospitals NHS Trust 2014

Patients in the Interventional arm (i.e. with Pleural Vent in situ) meeting the above can be discharged from hospital but return for daily review until completion of treatment. Patients in Control Arm (i.e. with chest drain in situ) must remain in hospital until full resolution of pneumothorax (see Section 8).

Note: The digital air leak measurements will be recorded on an e-CRF separate from those with other clinical information so that the responsible physician is unaware (blinded) of the electronic air leak measurement when making decision on discharge.

7.7. Follow-up Visits (All patients)

1 week post initial presentation to hospital (observational cohort)* or completion of treatment (randomised group) follow-up visit (+/- 1 day)

1 week after the Pleural Vent (Intervention arm) or chest drain (Control arm) was removed or the participant was discharged (observational cohort study), participants will be followed-up in outpatient clinic. They will undertake:

- CXR

  A repeat chest radiograph (preferably PA erect).

- CT scanning (1-2 weeks post completion of treatment)

  Computed Tomography (CT) scan* to detect lung parenchymal abnormalities (high resolution limited cuts apically) and airway inflammation (via scoring of bronchial wall thickness).

  Note: Patients who fail medical treatment (and hence referred for surgery, will have CT as an inpatient prior to any surgical intervention as is usual practice and therefore will not have an additional scan.

- CRF completion, review of patient resource diaries, quality of life assessments and VAS

  To document pneumothorax recurrence (ipsilateral or contralateral), need for surgery, prevalence of ongoing respiratory symptoms, duration of time off work and total healthcare expenditure (to be calculated on the basis of interaction with all healthcare services during the patient's primary episode and subsequent follow-up).

  **Please refer to the Trial Specific Instruction on Delayed Recruitment Follow-up for patients recruited after their initial presentation to hospital (days 1-14)(RAMPP_TSI_09_Delayed_Recruitment_Follow-up).*

1-month post initial presentation to hospital (observational cohort) or randomisation (randomised group) follow-up visit (not less than 30 days /+ 1 week)

- CXR

  A repeat chest radiograph (preferably PA erect).

- CRF completion, review of patient resource diaries, quality of life assessments and VAS

© Copyright: The University of Oxford and Oxford University Hospitals NHS Trust 2014

To document pneumothorax recurrence (ipsilateral or contralateral), need for surgery, prevalence of ongoing respiratory symptoms, duration of time off work and total healthcare expenditure (to be calculated on the basis of interaction with all healthcare services during the patient's primary episode and subsequent follow-up).

6-month post initial presentation to hospital (observational cohort) or randomisation (randomised group) follow-up visit (+/- 1 week)

- CXR

  A repeat chest radiograph (preferably PA erect).

- CRF completion, review of patient resource diaries, quality of life assessments and VAS

  To document pneumothorax recurrence (ipsilateral or contralateral), need for surgery, prevalence of ongoing respiratory symptoms, duration of time off work and total healthcare expenditure (to be calculated on the basis of interaction with all healthcare services during the patient's primary episode and subsequent follow-up).

12 month post initial presentation to hospital (observational cohort) or randomisation (randomised group) follow-up visit (+/- 1 month)

- CXR

  A repeat chest radiograph (preferably PA erect).

- CRF completion, review of patient resource diaries, quality of life assessments and VAS

  To document pneumothorax recurrence (ipsilateral or contralateral), need for surgery, prevalence of ongoing respiratory symptoms, duration of time off work and total healthcare expenditure (to be calculated on the basis of interaction with all healthcare services during the patient's primary episode and subsequent follow-up).

*CT Regime to be followed by local radiologist is a Low Dose CT scan of the Chest with 4 High-Resolution slices at the apices:

- Low Dose: Scan type helical full. Rotation time 0.5sec. Detector coverage 40mm. Helical thickness 0.625mm. Pitch 0.984:1. Speed 39.37. Kv 120. mA 60. Large FOV. Matrix 512x512. 1st Recon type chest ww 400/wl 40 recon type plus. DMPR set up to do - sag/cor 3mm. 2.5mm mediastinum axials and 5mm MIPS. 2nd Recon bone plus 512x512 recon type plus ww1500/wl-600 (This is for 2.5mm Axial reformats).

- HRCT: Scan type helical full. Rotation time 0.8sec. Detector coverage 1.25mm. Axial thickness and no. images per rotation 1.25mm/1. Kv 120. mA 200. Large FOV. 4 single slices at 10mm intervals down from the apex (starting at 10mm below the apex). Matrix 512x512. 1st Recon type bone plus ww 1500/wl.

A computer based algorithm will be developed to objectively assess CT scans using:

- Lung parenchymal density (in Hounsfield units, HU) - at 3 levels at the lung apices (both ipsilateral and contralateral lungs), with a reference density measurement of the lower lobes. A similar

© Copyright: The University of Oxford and Oxford University Hospitals NHS Trust 2014

method has been validated for use in patients with Chronic Obstructive Pulmonary Disease (COPD), with specific threshold value for emphysema (i.e. <-910 or <-950HU) [21]

- Presence, number and size of bullae at apices.
- Bronchial wall thickness in lung apices using digital measurement tool as surrogate for inflammation.

7.7.1. Failure to attend follow up

All patients will have out-patient follow-up at 1 week post completion of treatment (+/- 1 day), 1 month (+/- 1 week), 6 months (+/- 1 week) and 12 months (+/- 1 month) post enrolment to assess recurrence rate. Patients might receive text message reminders for their follow-up appointments. However, in exceptional circumstances, patients not able to attend the follow-up outcome point will be contacted by phone to at least check their status. If this is not possible as much data as possible should be collected through medical notes.

7.7.2. Patient Transfers

For patients moving from the area, every effort should be made for the patient to be followed-up (where possible this should be at another study site if available in the area). Alternatively, follow-up can occur by phone if needed.

7.7.3. Sample Handling

Patients will be asked to consent to have one blood sample taken (5ml) to be analysed for serum levels of highly sensitive C-reactive protein (CRP). This sample will be sent to ORTU for analysis. The remaining blood will be stored in the ORTU freezer, coded using trial number and initials as only patient identifiable information. They will only be accessible to members of the trial team and will later become part of the Biobank (the ORTU Collection of the Oxford Radcliffe Biobank (ORB)). Patients' samples may be used in future research, such as genetic factors predisposing to pneumothorax, provided patients consent to this and the samples will be gifted to the University of Oxford, again with patients' consent.

7.7.4. Discontinuation/Withdrawal of Participants from Study

In consenting to the study, patients are consenting to treatment according to the study protocol, follow-up and data collection. If a patient wishes to withdraw from the study, the investigator should nevertheless explain the importance of remaining in follow-up, or failing this of allowing routine follow-up data to be used for study purposes. If the patient explicitly states their wish not to contribute further data to the study, the patient should be withdrawn, the investigator should complete the withdrawal form as part of the e-CRF and the ORTU should be informed in writing. Data collected up to the point of withdrawal can still be included in the study or data collection can continue through medical notes as long as the patient agrees to this. If a patient requests full withdrawal, then samples will be destroyed and data not use.

7.7.5. Definition of End of Study

The study will close at the point when the last patient has completed their last follow-up visit.

8. INTERVENTIONS

© Copyright: The University of Oxford and Oxford University Hospitals NHS Trust 2014

Once the patient has been identified as having a PSP by chest radiograph (CXR) the decision to intervene will be made on the basis of the current BTS guidelines: Small (interpleural distance at level of hilum <2cm) and/or asymptomatic patients can be managed conservatively with observation according to trial specific instructions:

1. Observation cohort study:

1.1. Discharge home if meets "Fitness for Discharge" criteria (Section 7.6h) above.
1.2. Arrange for repeat CXR on Day 2 and Day 7 (+/- 1 day to allow for weekends).
1.3. Meet with research team after each CXR to assess resolution and symptoms.
1.4. Outpatient High resolution CT (HRCT) Chest between 1-2 weeks post discharge.
1.5. Follow-up at 1 week, 1, 6 and 12 months (post-completion of treatment) to assess recurrence rate.

Patients who do not require pleural intervention (aspiration or chest drain insertion) will still be followed up as part of the epidemiological component of the study.

Patients registered into the observational cohort who subsequently present with a pneumothorax which requires intervention (see below), at any time after enrolment, can be invited to take part in the treatment part of the study and randomised into one of the treatment arms. They will need to be consented and provided with a new trial number at randomisation. Their participation in the observational cohort part of the study would then cease and they would be followed up as per their newly assigned treatment arm (see 7.6. Study Assessments). Patients transferring form the observational cohort into one of the treatment arms will be consented to have their bloods taken at randomisation to measure HS CRP (but no further samples will be taken for genetic analysis).

Any patient requiring intervention, i.e. a large (interpleural distance at level of hilum >2cm) and/or symptomatic pneumothorax will be randomised to one of two management arms (see flow diagram in appendix 1):

2. Intervention Arm: Placement of an apical ambulatory chest drainage device (Rocket Pleural Vent)

2.1. Ambulatory device should be inserted immediately after randomisation, using local anaesthetic. Researchers and local clinicians will be trained in inserting the device (see Trial Specific Instructions (TSI) for detail – these will be developed before the start of the study and stored in the Investigational Site File).
2.2. Observe to check for clinical stability (1-2 hours).
2.3. Repeat CXR after observation period.
2.4. If CXR shows sufficient re-expansion of lung† and no ongoing air leak clinically††, remove device and patient can be discharged. As standard practice, a post-removal CXR should be performed to ensure that the lung has not re-collapsed.
2.5. If CXR shows insufficient re-expansion of lung†, the Pleural Vent should remain in place and the patient can be discharged with device in situ if fulfils “Fitness for discharge” criteria (see Section 7.6h). Once discharged patients should be seen every 1-2 days until day 4: weekdays on the respiratory ward, respiratory outpatient clinic, or Day Case Unit (will vary according to local site facilities) by the researcher; weekend review on the Respiratory ward by the on-call SpR or consultant.
2.6. If CXR shows no significant improvement and the patient remains breathless, the patient should be admitted to the Respiratory Ward and reviewed daily with repeat CXR.

© Copyright: The University of Oxford and Oxford University Hospitals NHS Trust 2014

2.7. Once admitted, if CXR shows resolution, no pneumothorax and no air leak, the device should be removed and the patient discharged. As standard practice, a post-removal CXR should be performed to ensure that the lung has not re-collapsed.

2.8. Once admitted, if insufficient re-expansion of lung† and / or air leak†† – assess clinical stability and mobility. If fulfils "Fitness for discharge" criteria, discharge home with the device in situ with plan for daily review (as above).

2.9. High resolution CT (HRCT) Chest should be performed as an outpatient between 1-2 weeks post completion of treatment or as an in-patient if referred for surgery (see section 7.6)

2.10. All patients will have out-patient follow-up at 1 week post completion of treatment and at 1, 6 and 12 months post enrolment to assess recurrence rate.

† "Sufficient re-expansion" is defined as complete or almost complete re-expansion (only a very small (<1cm) rim of air apically) on CXR.
†† On going air leak assessed by attempted aspiration through the device using a syringe and connector: if device patent (as assessed by movement of the in-built diaphragm) but unable to aspirate (i.e. draw air back through syringe) then there is no ongoing air leak; if able to aspirate air freely, then there is ongoing leak and active pneumothorax.

Only clinicians trained in the Rocket Pleural Vent use and insertion as part of the trial will be permitted to insert it using the appropriate TSI and training provided for both the standard care and ambulatory arms.

3. Control Arm: Standard management as per BTS guidelines:

3.1. Pleural aspiration, if clinician deems appropriate, should be attempted under local anaesthetic using 14-16 gauge cannula and syringe. Not more than 2.5L should be aspirated

3.2. Repeat CXR after aspiration.

3.3. Observe for 1-2hours to check for clinical stability.

3.4. Repeat CXR after observation period: if sufficient re-expansion of lung† patient can be discharged home.

3.5. If CXR (either after initial aspiration or repeat after observation period) shows insufficient re-expansion, then a small-bore chest drain (< or =14F) should be inserted and attached to an underwater seal.

3.6. Admit the patient: either to Acute Medical Ward or, ideally, to Respiratory ward. If not admitted directly to respiratory ward, arrangements should be made to transfer them as soon as a bed becomes available.

3.7. Clinician may decide to proceed directly to chest drain insertion and admission at their discretion (and according to BTS guidelines).

3.8. Decisions regarding drain removal are as per the BTS guidelines and standard practice at the participating centre (including no further air leak as demonstrated by a non-bubbling chest drain), and full lung expansion on chest x-ray. As standard practise, a post-removal CXR should be performed to ensure that the lung has not collapsed.

3.9. "Fitness for Discharge" criteria for discharge will be conducted daily to provide equality between treatment arms (criteria as above).

3.10. High resolution CT (HRCT) Chest should be performed between 1-2 weeks post completion of treatment: either as an inpatient or as an outpatient if already discharged.

3.11. All patients will have out-patient follow-up at 1 week post chest drain removal and at 1, 6 and 12 months post enrolment to assess recurrence rate.

© Copyright: The University of Oxford and Oxford University Hospitals NHS Trust 2014

† "Sufficient re-expansion" is defined as complete or almost complete re-expansion (only a very small (<1cm) rim of air apically) on CXR.

Late Enrolment

Ideally, all patients will be identified and considered for entry into the trial on their initial assessment, either by A&E staff, general medical team or respiratory team. However, in order to maximise recruitment, patients requiring intervention (including patients who have undergone an initial treatment procedure) can be consented up to 24 hours after initial presentation to hospital.

Patients who had an initial aspiration (as per BTS guidelines) without full re-expansion, can be enrolled and randomised.

Patients who have already had a chest drain inserted but still have residual pneumothorax and on-going air leak (bubbling) can also be consented, but their consent process would need to specifically reflect the two subsequent scenarios:

- Patients subsequently randomised to Control Arm, would be entered into the trial using the existing chest drain.
- Patients randomised to Intervention Arm would need to have the ambulatory device inserted and then the existing chest drain removed.

Patients would need to be aware of this potential additional procedure, but the benefit to the patient is that, if randomised to the Intervention (ambulatory) arm, they have the potential to be managed as an outpatient (if discharge criteria are met, as described in section 7.5). All such issues will be openly discussed with the patient.

Patients who did not require intervention can be invited to participate in the observation cohort study, up to 2 weeks after presentation. Baseline information will be gathered from patient's medical notes. Observational cohort patients enrolled late may need to follow a modified follow-up schedule (e.g. if enrolled after 1 Week follow-up was due, their first follow-up visit would be 1 Month post completion of treatment).

9. SAFETY REPORTING

9.1. Definition of Adverse Event (AE) and Serious Adverse Event (SAE)

Adverse Event (AE) - any untoward medical occurrence in a clinical trial subject.

Adverse Device Effects (ADEs) - untoward and unintended medical occurrences in response to a medical device.

All cases judged by either a medically qualified professional or the sponsor as having a reasonable suspected causal relationship to the device qualify as a device effect. This also includes any event resulting from insufficiencies or inadequacies in the instruction for use or deployment of the device and includes any event that is a result of a user error.

Serious Adverse Event (SAE)- any untoward medical occurrence that:

- Results in death,
- Is life-threatening,

© Copyright: The University of Oxford and Oxford University Hospitals NHS Trust 2014

- Requires inpatient hospitalisation or prolongation of existing hospitalisation,
- Results in persistent or significant disability/incapacity, or
- Is a congenital anomaly/birth defect,
- Other important medical events*.

*Other events that may not result in death, are not life threatening, or do not require hospitalisation, may be considered a serious adverse event when, based upon appropriate medical judgement, the event may jeopardise the participant and may require medical or surgical intervention to prevent one of the outcomes listed above.

Serious adverse events in this study which should be reported immediately (i.e. within 24 hours) to ORTU include:

- Tension pneumothorax occurring during treatment (until discharge)
- Blockage of drain with clinical consequences (e.g. patient unwell, further procedure)
- Major haemorrhage which requires specific intervention (e.g. blood transfusion)
- Any additional emergency pleural procedure as deemed necessary by the responsible local physician (e.g. large bore chest drain insertion)

A Serious Adverse Device Effect (SADE) - any untoward medical occurrence seen in a patient that can be attributed wholly or partly to the device which resulted in any of the characteristics or lead to the characteristics of a Serious Adverse Event.

A SADE is also any event that may have led to these consequences if suitable action had not been taken or intervention had not been made or if circumstances had been less opportune. A SADE will be documented on an SAE form and reported immediately (i.e. within 24 hours) to ORTU.

Unanticipated Adverse Device Effect (UADE) - Serious Adverse Device Effect that has not previously been identified in nature, severity or degree of incidence in the investigational plan or application (including a supplementary plan or application).

9.2. Expected Adverse Events

Expected adverse events are those events which are expected according to what is already documented in any reference documents for events associated with the trial interventions.
The following are considered to be expected adverse events associated with the proposed trial interventions for this trial:

- Pain at drain site
- Minor haemorrhage
- Subcutaneous emphysema
- Pleural infection
- Unintentional removal ("falling out")
- Recurrence of pneumothorax/worsening of ongoing pneumothorax (if no evidence it fully resolved)
- Re-expansion pulmonary oedema

© Copyright: The University of Oxford and Oxford University Hospitals NHS Trust 2014

- Any further (non-emergency) pleural procedure required

9.3. Recording and Reporting Procedures

9.3.1. Events occurring prior to chest drain/Rocket Pleural Vent removal

All AEs/ADEs which occur until drain/Rocket Pleural Vent removal, including those expected events listed above, should be recorded on the relevant e-CRFs. A separate AE form should be completed for any unexpected reaction or event not listed in the protocol and e-CRFs. Should any of the recorded events meet the criteria for an SAE/SADE, they should also be recorded on an SAE form and reported to the trial team at the ORTU. Reporting of SAEs should occur within **24 hours** of the local trial team becoming aware of the event.

9.3.2. Events occurring after completion of treatment

After chest drain/Rocket Pleural Vent removal, only those AE potentially related to the trial interventions (chest drain or Rocket Pleural Vent insertion/removal) or those considered of relevance to the trial should be recorded. The events should be recorded on the relevant e-CRF with a separate AE form completed for any events not listed as expected in the protocol and e-CRFs.

Any event occurring after chest drain/Rocket Pleural Vent removal which meets the criteria for an SAE/SADE should be discussed with the local principal investigator. If, in their opinion, there is a reasonable possibility that the event is related to the trial intervention, or if the event is of particular medical interest, it should be recorded on an SAE form and reported to the trial manager at ORTU, Oxford. Reporting of SAEs should occur within **24 hours** of the local trial team becoming aware of the event.

Recurrence of pneumothorax is expected in approximately 33% of PSP within 1 year. This recurrence information will explicitly be captured on the eCRFs. Therefore, recurrence events i.e.

- recurrence of pneumothorax (if fully resolved and documented as fully resolved) or
- worsening of their ongoing pneumothorax (if discharged but no evidence that it fully resolved)

do not need to be reported as an SAE (even if they meet criteria of requiring hospitalisation or prolongation of their hospital stay). All the information regarding these events should be recorded on the follow-up eCRFs.

Other events which meet the criteria for an SAE, but which are not felt by the PI to be of relevance to the trial need not be reported.

For patients in the observational cohort, only those AEs/SAEs which are of relevance to the trial as judged by the local PI should be recorded/reported.

9.3.3. Reporting to REC

Serious adverse events (including adverse defects) that in the opinion of the principal investigator or the Chief Investigator are: ‘related’ and ‘unexpected’ – i.e. the type of event is not listed in the protocol or brochure information for the device as an expected occurrence, will be reported to the REC that gave a favourable opinion of the study. Reports of related and unexpected SAEs will be submitted within 15

© Copyright: The University of Oxford and Oxford University Hospitals NHS Trust 2014

days of the Chief Investigator becoming aware of the event, using the NRES report of serious adverse event form. This will be coordinated by the ORTU team.

9.3.4. Following reporting

All reported events should be followed to resolution, including those which lead to withdrawal from the trial. The decision to withdraw a patient from the trial due to an adverse event rests with the principal investigator. Should a patient request withdrawal, outcome data will still be gathered unless consent for this is also withdrawn.

9.4. Ambulatory Device (Rocket Pleural Vent) Safety Testing

Rocket Pleural Vent is CE marked and has been in clinical use in the UK for the ambulatory management of pneumothorax since September 2014. The Oxford Respiratory team are planning their use in patients post-thoracoscopy, and they have been used in two UK centres successfully (Leicester and Cambridge).

10. STATISTICS AND ANALYSIS

10.1. Number of Participants

The primary outcome measure will be total length of stay in hospital to include primary hospital stay and re-admissions up to 30 days post randomisation. 30 days has been chosen on the basis of previous data suggesting that the majority of conservatively treated (non-surgical) air leaks will have resolved within 14 days of initial treatment in pneumothorax, and a 30 days outcome point is therefore conservative and will reliably capture all related re-admissions. Re-admission will be defined as the requirement of emergency (non-planned) visit to hospital requiring any form of contact with medical services (not restricted to further pleural interventions) in relation to the pneumothorax. This will not include planned day case reviews for the outpatient treated population as specified in the application.

Justification of sample size - Primary outcome

The difference to detect is 2.3 days: from a mean of 4 days admission in the control arm to an expected mean of 1.7 days in the intervention arm, (Standard deviation in both groups 6.0). It is assumed, conservatively that ~20% of patients in the intervention arm will require a re-admission. Therefore, to detect this difference accounting for non-parametric data requires 236 patients in total, including a 10% attrition rate (80% power, 5% 2 sided significance). Our previous studies have demonstrated an attrition rate for the primary outcome measure of <5%.

Predictive model development (epidemiological component)

One of the secondary outcomes of the study is to determine whether digitally measured air leak and its evolution over treatment can predict short term clinical trajectory in patients with pneumothorax, including requirement for prolonged drainage and need for thoracic surgical intervention. Following consultation with the Centre for Statistics in Medicine on predictive model studies, a minimum of 10 events per variable are required to formulate predictive criteria robustly. There are expected to be 33% patients with unresolved pneumothorax at 7 days and 20% surgical requirement as in-patients on the basis of previous data. Data from 120 patients would permit the robust assessment of 4 variables as

© Copyright: The University of Oxford and Oxford University Hospitals NHS Trust 2014

potential predictive parameters: these are air leak at day 0, day 1 and day 2 and evolution of air leak (change from day 0 to 2). Therefore, the target recruitment target of 236 would be more than sufficient.

The predictive model for need for surgical referral will be developed by analysing the statistical significance of each parameter using linear-regression (when using the continuous variable of amount of air leak) and logistic-regression models (when considering a threshold level of air leak, e.g. >100ml/min, <100ml/min), corrected for baseline differences of statistical significance and biological plausibility. The epidemiological data of demographics, pain and breathlessness scores and recurrence rates will be collated but not specifically used in the predictive model.

The study will continue to recruit until 236 patients have been recruited into the randomised trial. The concurrent observational cohort (of patients managed conservatively) will continue to enrol patients until this time.

10.2. Analysis of Endpoints

All primary and secondary outcomes will be carried out on an intention to treat basis (i.e. patients will be analysed according to their randomised treatment arm, irrespective of what treatment they received).

10.2.1. Analysis of primary outcome

The primary outcome will be analysed using the Mann-Whitney U test. The median hospital stay will be reported for each arm and the 95% confidence interval for difference in medians will be calculated using an exact test.

As a sensitivity analysis survival analysis techniques will be used. Kaplan-Meier survival curves will be presented graphically. Survival will be compared between arms using the Gehan-Breslow-Wilcoxon test, which is more appropriate than the log-rank test when the rate of early events is high. Cox proportional hazards regression will also be used to calculate the hazard ratio and 95% confidence interval.

10.2.2. Analysis of secondary outcomes

Continuous secondary outcome measures will be analysed using analysis of covariance adjusting for baseline score. Results will be reported as adjusted mean difference between treatment arms, with 95% confidence interval and p-value. Categorical secondary outcome measures will be analysed using the chi-squared test (or Fisher's exact test when cell sizes are small (i.e. less than 5)). Time to event secondary outcome measures will be analysed in the same way as the primary outcome measure.

At the conclusion of the study, we will use a reproducible CXR scoring system to assess the size of the pneumothoraces. The system was originally used to measure the size of pleural effusion, developed during the MIST2 trial [22]. This will be used to quantify the size of the pneumothorax as follows: For each image, the areas occupied by the pneumothorax and by the hemithorax are manually drawn around to form a polyhedron. The polyhedron areas are saved separately as JPEG files and the percentage area of each polyhedron is calculated using pixel counting Image J software (available online).

Patient related factors and time to return to work will be collated blindly and verified by a central panel, unaware of interventional arm and/or requirement of surgery.

© Copyright: The University of Oxford and Oxford University Hospitals NHS Trust 2014

Data collected on air leak by digital suction device (Thopaz), and on CT imaging will be assessed against these objective criteria.

10.2.3. Cost-effectiveness analysis

Health economic outcomes

The perspective adopted in the economic analysis will be that of the UK National Health Service. As a result we will collect information on the following resource use items over the 12 month follow-up:

1. The costs of providing the two interventions under study. This will entail collecting information on theatre time, staff time, consumables, and any diagnostic tests. In addition, an average cost per procedure will be estimated by direct observation of a sample of procedures undertaken in each patient group.

2. Initial and subsequent hospitalisation.

3. Outpatient contacts. This will entail collecting information on patient's use of resources after discharge from hospital, including contacts with outpatient services, use of emergency departments and emergency transport.

Information on 2) and 3) will be obtained by reviewing the administrative care records in each of the participating centres using pre-defined questionnaires to be filled in by study staff.

4. Primary care contacts. This will include any surgery or home visits by general practitioners and community/district nurses. Information will be obtained using a patient questionnaire designed to collect information in the trial. This questionnaire will be administered by study staff as part of all the follow-up interviews at 1 week and 1 and 6 months. To aid patients in their recall process, patients will be supplied at the start of the trial with specially designed patient diaries.

Resource use items will be priced using unit cost schedules such as PSSRU, British National Formulary and NHS Reference costs. If necessary, finance departments at each of the study centres will be contacted to obtain unit cost information not included in these sources.

As the main outcome measure in the economic evaluation will be incremental cost per Quality-Adjusted Life Year (QALY) gained, generic quality of life information will be collected. In line with the recommendations from the National Institute for Health and Clinical Excellence (NICE), the EuroQol EQ-5D-5 levels – a widely used generic multi-attribute utility scale – will be completed for each patient at baseline, 1 week, 1 and 6 month assessments to measure patients' general health related quality of life. For QALY construction, EQ-5D-5L results will be translated into utility values using published UK population valuations.

Health economic analysis

An economic evaluation, adherent to guidelines for good economic evaluation practice, will be undertaken integral to the main trial. A within-trial cost-utility analysis will explore the incremental cost per QALY gained of ambulatory care when compared to standard care. Cost and effect results will be reported as means with standard deviations, with mean differences between the two patient groups reported alongside 95% confidence intervals. Depending on the amount of missing cost and quality of life data, missing data will be imputed using recommended multiple imputation methods, with results from

© Copyright: The University of Oxford and Oxford University Hospitals NHS Trust 2014

this analysis being presented as an additional sensitivity analysis. Incremental cost-effectiveness will be calculated by dividing the difference in costs by the difference in effects. Uncertainty around the incremental cost-effectiveness ratio (ICER) will be explored using non-parametric bootstrapping. All cost-effectiveness results will be presented on the cost-effectiveness plane and as cost-effectiveness acceptability curves, indicating where the results fall in relation to a given cost-effectiveness threshold.

10.3. Interim Monitoring and Analyses

A blinded interim analysis of the primary outcome (hospital stay) will be undertaken after approximately 50% patients have been recruited in order to assess the assumptions made in the sample size calculation. This analysis will be reviewed by the DSMC who will make recommendations regarding any necessary changes to the sample size required. No correction of the significance level of the final analysis is planned on this single assessment of early event rate by the DSMC.

Interim analysis of the secondary outcomes (including air leak measurement, duration of treatment, assessment of CT scans and recurrence rates) will be conducted at 50% recruitment. No analysis of the treatment allocation will be undertaken, so the data will remain blinded. These data will be used as part of the Doctoral (PhD) thesis for Dr R Hallifax (Trial Coordinator) and may be published in advance of the full trial results as a “derivation” dataset. The remaining dataset could then be used to “validate” the findings from the interim analysis, in order to maximise the impact of the results to the public and wider medical community.

10.4. Analysis Plan (Summary)

A full analysis plan will be developed, agreed and signed off prior to locking the database and any data analysis as per OCTRU SOPs (see statistical analysis above).

10.5. Measures of compliance and adherence

Compliance with the trial protocol will be specifically assessed for the outcome measures. Objective criteria for these outcome measures will be documented within the e-CRFs. Fulfilling these criteria (e.g. for surgery) does not require the patient to be referred for surgery, but will be used in analysis as a secondary outcome point to assess patients potentially eligible for surgery during the study.

11. DATA MANAGEMENT

11.1. Access to Data

Direct access will be granted to authorised representatives from the Sponsor including members from the CTU or host institution for monitoring and/or audit of the study to ensure compliance with relevant regulations and SOPs.

11.2. Data Recording and Record Keeping

11.2.1. Database

© Copyright: The University of Oxford and Oxford University Hospitals NHS Trust 2014

This study will utilise a web-based, trial database (OpenClinica). OpenClinica is a dedicated and validated clinical trial database designed for remote electronic data capture. The Chief Investigator will act as Data Custodian for the trial. A guide explaining how to use OpenClinica will be provided to every site. Relevant ORTU staff will have overview of all entered data.

The study database is bespoke and hosted on the University of Oxford server with services provided through Oxford University Medical Sciences Division IT Services (MSD IT). The server and database are protected by a number of measures including anti-virus and anti-spyware applications, firewalls, encryption technology and permissions. The database will be backed up on a daily basis.

The database and access to computers are password protected. Paper-based identifiable data at each site will be kept in a locked cabinet, in a locked or ID-access controlled area. The Data Manager will maintain a list of personnel to grant and revoke access.

11.2.2. Data Entry and Query Management

Patients recruited into the study are identified by their Trial Number and their initials. Sites enrolling patients will access the database through a secure weblink. The database is secure and password-protected. Any person entering data (usually respiratory clinicians and research nurses and/or staff at A&E departments) at local sites will have access to patients enrolled at that site only and will require their own unique log-in to access the database, with a system-generated password that can be changed at first log-in. Each individual user will have specified permissions and authorisations at their local site (e.g. Investigator, Data Entry). Data will be entered into the electronic CRF and saved directly to the central clinical database outlined above (apart from the EQ-5D-5L and VAS questionnaire which will be completed by patients on paper first and then entered onto the electronic database). Only the Site Investigator will have the access and ability to sign-off e-CRFs locally. Every activity at a local site will be recorded on the database as part of the audit trail. The study Data Manager and Data Co-ordinator will perform quality checks of data entered and also assist with site training.

The data stored will be checked for missing or unusual values and for consistency within participants over time. If any problems are identified, the appropriate e-CRFs will be reviewed in discussion with relevant local site personnel and queried for confirmation or correction as required until resolution. Should any data require changing, this will be electronically tracked (name of reviewer, changes made and date) for the purposes of any future audit or external review.

Any patients initially enrolled into the observational cohort study who subsequently require treatment will be invited to take part in the treatment part of the study. Once consented, they will be randomised into one of the treatment arms and assigned a new trial number and any further data collected will be under the new trial number. A log will be created so as to not double-count the demographics of these patients in subsequent analyses.

Patient images (CXRs and CT scan) will be uploaded electronically through a secure weblink to the trial database (OpenClinica) at the completion of treatment and subsequently at follow-up clinic visits.

11.2.3. Data Quality and Security

The data will be securely stored in line with the principles of GCP standards and the data protection Act. Standard Operating Procedures (SOPs) will be followed to ensure quality control. Data validation of primary data will include at least confirmation of participant identity, informed consent, eligibility criteria

© Copyright: The University of Oxford and Oxford University Hospitals NHS Trust 2014

and primary outcome data; this validation process will be carried out by the study team in a subset of participants (approximately 10%).

The Chief Investigator and authorised staff based at the ORTU will have access to participants' data from across all sites. The Principal Investigators at individual site will facilitate access to study records for the purpose of monitoring, audits, and regulatory inspections. Participant's consent to this will be sought at the time of enrolment into the study.

Minimising missing data

In order to maximise the completion of follow-up data, research staff at individual sites may contact patients to remind them of follow-up appointments and to provide support. Additionally, patients can consent to receiving text or email alerts and updates regarding their follow-up plan. The Data Manager or delegate will chase the sites for missing e-CRFs and questionnaires on a monthly basis.

12. QUALITY ASSURANCE PROCEDURES

ORTU has extensive experience in managing large multi-centre (>1000 patients in the last 8 years) and has an established track record of delivering high quality trials with excellent follow-up rates.

The study will be monitored, or audited in accordance with the current approved protocol, GCP, relevant regulations and standard operating procedures. A detailed Risk Assessment will be conducted before the trial starts and an appropriate Monitoring Plan will be developed by ORTU.

12.1. Study infrastructure

12.1.1. Trial Management Group (TMG)

The TMG is responsible for the day-to-day management of the study. The TMG is responsible for all aspects of the study (including recruitment rate, budget management, protocol compliance etc.) and for ensuring appropriate action is taken to safeguard study participants and the quality of the study. The TMG will comprise of the study Chief Investigator, Study Clinical Co-ordinator, Trial Manager, Data Manager, Trial Statistician and a Research Nurse.

12.1.2. Trial Steering Committee (TSC)

The TSC consists of both independent members as well as researchers working on the study. The role of the TSC is to provide overall supervision of the study and monitor the progress of the study to ensure it is being conducted in accordance with the protocol, relevant regulations and the principles of GCP. The TSC will meet at regular intervals (as per the TSC charter) and will comprise of an Independent Chairperson, Chief Investigator, Study Clinical Co-ordinator, Study Manager, Independent Statistician and other Independent Members including Patient Representative.

12.1.3. Data Safety Monitoring Committee (DSMC)

The Data Safety Monitoring Committee consists of independent experts (medical experts and a Statistician) external to a trial who assess the progress, conduct, participant safety and critical endpoints of a clinical trial. The DSMC will meet on a regular basis as specified in the DSMC charter to review the study information and accruing data during the conduct of the study and make recommendations to the TSC.

© Copyright: The University of Oxford and Oxford University Hospitals NHS Trust 2014

13. ETHICAL AND REGULATORY CONSIDERATIONS

Each patient's consent to participate in the study should only be sought and obtained after a full explanation of the study's nature has been given (including provision of the PIL), including discussion of the proposed physiological measurements and randomised nature of the ambulatory treatment protocol. The right of the patient to refuse to participate in the study without giving any reason must be respected. As pneumothorax is an acute medical problem, it would not be appropriate to wait the usual 24 hours to allow patients time to read the patient information leaflets, prior to intervention. Therefore, ideally the patient should still be given a reasonable short period of time to read, digest and ask questions about the study, prior to an approach for consent.

13.1. Declaration of Helsinki

The Investigator will ensure that this study is conducted in accordance with the principles of the Declaration of Helsinki.

13.2. Guidelines for Good Clinical Practice

The Investigator will ensure that this study is conducted in accordance with relevant regulations and with Good Clinical Practice.

13.3. Approvals

The protocol, informed consent form, participant information sheet, GP letter and any proposed advertising material will be submitted to an appropriate Research Ethics Committee (REC), and host institution(s) for written approval.

The trial will open to recruitment following a documented Green Light Process by the Oxford Clinical Trials Research Unit (OCTRU).

The Investigator will submit and, where necessary, obtain approval from the above parties for all substantial amendments to the original approved documents.

13.4. Reporting

The CI shall submit once a year throughout the study, or on request, an Annual Progress report to the REC Committee, host organisation, Funder and Sponsor. In addition, an End of Study notification and final report will be submitted to the same parties.

13.5. Participant Confidentiality

Staff involved in the study will ensure that all participants' confidentiality is maintained. The participants will be identified only by a unique trial number on the e-CRF and clinical trial database. All documents will be stored securely and only accessible by staff involved in the study and authorised personnel. Any personal information such as phone numbers etc. collected for the purpose of follow-up will only be accessible to the local research teams. The study will comply with the Data Protection Act and other relevant local legal requirements alongside the principles of ICH-GCP. The coded study data will be stored for at least five years following closure of the study and thereafter disposed of in line with

© Copyright: The University of Oxford and Oxford University Hospitals NHS Trust 2014

regulatory requirements. No participant will be individually identified in any subsequent publications relating to this study.

13.6. Expenses and Benefits

Reasonable travel expenses for any visits additional to normal care will be reimbursed on production of receipts, or a mileage allowance provided as appropriate.

13.7. Other Ethical Considerations

It is possible that whilst undergoing investigation as part of their participation in this study, a participant may have an incidental abnormality identified. In these cases, the participant will be counselled immediately by the investigator and referred on for further clinical investigation as deemed appropriate and agreed. Another ethical consideration is the relatively short period of time to fully consider the information prior to consent, as intervention (if required) should be delivered without delay. Patients enrolled after already having had a chest drain (late recruitment), will require an extra procedure (device insertion and chest drain removal) if they are randomised to the ambulatory arm and some may therefore decide not to take part.

14. FINANCE AND INSURANCE

14.1. Funding

This study is funded by a Research for Patient Benefit grant from the National Institute of Health Research and a Medical Research Council Training Fellowship for the Trial Coordinator (Dr Rob Hallifax).

14.2. Insurance

The University has a specialist insurance policy in place which would operate in the event of any participant suffering harm as a result of their involvement in the research (Newline Underwriting Management Ltd, at Lloyd's of London). NHS indemnity operates in respect of the clinical treatment which is provided.

15. PUBLICATION POLICY

The preparation of a manuscript for rapid publication will be a priority for and sole responsibility of the Trial Management Group, under the overall supervision of the Chief Investigator. The Trial Management Group will also take responsibility for reviewing drafts of any manuscripts, abstracts, press releases and other publications arising from this study. It is anticipated that an initial report would be completed within six months of the study's closure. The Trial Management Group will approve a definitive manuscript detailing the final overall results of the study. Raw data from the study will be made accessible to the public on request once the study has been completed and final results been published.

All publications will include a list of investigators, and named authors will include the study's Chief Investigator, Key Investigator, Statistician and Trial Manager as a minimum. Authors will be determined in accordance with ICMJE guidelines and other contributors to the study will be acknowledged. Authors will acknowledge that the study has been sponsored by the University of Oxford, UK.

© Copyright: The University of Oxford and Oxford University Hospitals NHS Trust 2014

© Copyright: The University of Oxford and Oxford University Hospitals NHS Trust 2014

16. REFERENCES

[1] Melton LJ, Hepper NGG, Offord KP, " Incidence of spontaneous pneumothorax in Olmsted County, Minnesota: 1950 to 1974.," *Am Rev Respir Dis,* vol. 102, pp. 1379-82, 1979.

[2] Schoenenberger, R. A., W. E. Haefeli, et al, "Timing of invasive procedures in therapy for primary and secondary spontaneous pneumothorax," *Archives of Surgery,* vol. 126, no. 6, pp. 764-766, 1991.

[3] Schramel FM, Postmus PE, Vanderschueren RG, "Current aspects of spontaneous pneumothorax," *Eur Respir J,* vol. 10, no. 6, pp. 1372-9, 1997.

[4] MacDuff A, Arnold A, Harvey J, "Management of spontaneous pneumothorax: British Thoracic Society Pleural Disease Guideline 2010," *Thorax,* vol. 65, no. Suppl 2, pp. ii18-31, August 2010.

[5] Bense L, Lewander R, Eklund G, Hedenstierna G, Wiman LG., "Nonsmoking, non-alpha 1-antitrypsin deficiency-induced emphysema in nonsmokers with healed spontaneous pneumothorax, identified by computed tomography of the lungs.," *Chest. 1993,* vol. 103, no. 2, pp. 433-8, 1993.

[6] Amjadi K, Alvarez GG, Vanderhelst E, Velkeniers B, Lam M, Noppen M., "The prevalence of blebs or bullae among young healthy adults: a thoracoscopic investigation.," *Chest. 2007,* vol. 132, no. 4, pp. 1140-5, 2007.

[7] Noppen M, Dekeukeleire T, Hanon S, Stratakos G, Amjadi K, Madsen P, Meysman M, D'Haese J, Vincken W., "Fluorescein-enhanced autofluorescence thoracoscopy in patients with primary spontaneous pneumothorax and normal subjects," *Am J Respir Crit Care Med,* vol. 174, no. 1, pp. 26-30, 2006.

[8] Smit HJ, Golding RP, Schramel FM, Devillé WL, Manoliu RA, Postmus PE., "Lung density measurements in spontaneous pneumothorax demonstrate airtrapping," *Chest,* vol. 125, no. 6, pp. 2083-90, 2004.

[9] Lichter I, Gwynne JF, "Spontaneous pneumothorax in young subjects. A clinical and pathological study.," *Thorax,* vol. 26, no. 4, pp. 409-17, 1971.

[10] Mercier C, Page A, et al, "Outpatient management of intercostal tube drainage in spontaneous pneumothorax," *Annals of Thoracic Surgery ,* vol. 22(2), pp. 163-165, 1976.

[11] Dernevik L, Roberts D, Hamraz B, Nordstrand-Myntevik M, "Management of pneumothorax with a mini-drain in ambulatory and hospitalized patients," *Scand Cardiovasc J,* vol. 37(3), pp. 172-6., 2003.

[12] Verma A, Tee A, et al, "The impact of Heimlich valve on the treatment of primary spontaneous Pneumothorax," *Respirology ,* vol. 14, p. A167 , 2009.

[13] Choi S, "Can spontaneous pneumothorax patients be treated by ambulatory care management?," *European Journal of cardio-thoracic surgery,* vol. 31, pp. 491-95, 2007.

[14] Ponn RB, Silverman HJ, et al, "Outpatient chest tube management," *Annals of Thoracic Surgery,* vol.

© Copyright: The University of Oxford and Oxford University Hospitals NHS Trust 2014

64(5), pp. 1437-1440, 1997.

[15] Hassani B, Foote J, Borgundvaag B,, “Outpatient management of primary spontaneous pneumothorax in the emergency department of a community hospital using a small-bore catheter and a heimlich valve,” *Academic Emergency Medicine,* vol. 16, pp. 513-8, 2009.

[16] Marquette CH, Marx A, et al, “Simplified stepwise management of primary spontaneous pneumothorax: A pilot study,” *European Respiratory Journal,* vol. 27(3), pp. 470-476 , 2006.

[17] Cannon WB, Mark JBD, et al, “Pneumothorax: A therapeutic update,” *American Journal of Surgery,* vol. 142(1), pp. 26-29, 1981.

[18] Campisi P, Voitk J, “Outpatient treatment of spontaneous pneumothorax in a community hospital using a Heimlich flutter valve: A case series,” *Journal of Emergency Med,* vol. 15, pp. 115-9, 1997.

[19] Lai SM, Tee AK, “Outpatient treatment of primary spontaneous pneumothorax using a small-bore chest drain with a Heimlich valve: the experience of a Singapore emergency department,” *Eur J Emerg Med,* vol. 19, no. 6, pp. 400-4, 2012.

[20] Brims FJ, Maskell NA., “Ambulatory treatment in the management of pneumothorax: a systematic review of the literature,” *Thorax,* p. [Epub ahead of print], March 2013.

[21] Gierada DS, Yusen RD, Pilgram TK, Crouch L, Slone RM, Bae KT, Lefrak SS, Cooper JD, “Repeatability of quantitative CT indexes of emphysema in patients evaluated for lung volume reduction surgery,” *Radiology,* vol. 220, p. 448–454, 2001.

[22] Rahman NM1, Maskell NA, West A, Teoh R, Arnold A, Mackinlay C, Peckham D, Davies CW, Ali N, Kinnear W, Bentley A, Kahan BC, Wrightson JM, Davies HE, Hooper CE, Lee YC, Hedley EL, Crosthwaite N, Choo L, Helm EJ, Gleeson FV, Nunn AJ, Davies RJ., “Intrapleural use of tissue plasminogen activator and DNase in pleural infection,” *N Engl J Med,* vol. 365, no. 6, pp. 518-26, Aug 2011.

© Copyright: The University of Oxford and Oxford University Hospitals NHS Trust 2014

17. APPENDIX A: STUDY FLOW CHART

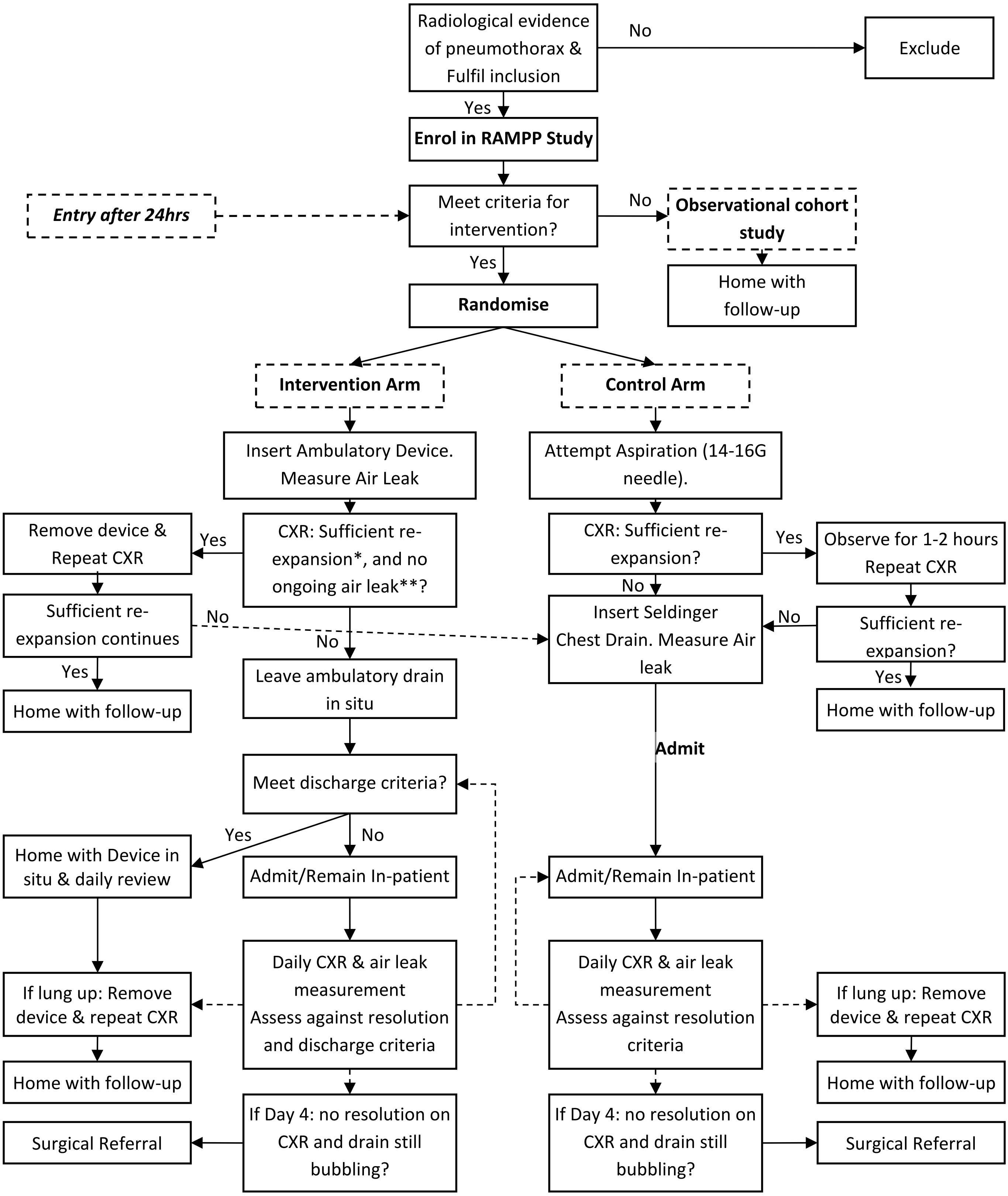

* "Sufficient re-expansion" is defined as complete or almost complete re-expansion (only a very small (<1cm) rim of air apically) on CXR.

** On going air leak assessed by attempted aspiration through the device using a syringe and connector: if device patent (moving in-built diaphragm) but unable to aspirate then there is no ongoing air leak; if able to aspirate air freely, then there is ongoing leak and active pneumothorax.

© Copyright: The University of Oxford and Oxford University Hospitals NHS Trust 2014

18. APPENDIX B: SCHEDULE OF STUDY PROCEDURES

Study Procedures	Baseline	Post randomisation (Intervention and control arms only)	Days 0-4 (Intervention and control arms only)	Day 2 (Observational Cohort study only)	Day ≥4	Completion of treatment (Intervention and control arms only)	1 week post initial presentation/ completion of treatment follow-up (All patients)	1 month post initial presentation/ randomisation follow-up (All patients)	6 months post initial presentation/ randomisation follow-up (All patients)	12 months post initial presentation/ randomisation follow-up (All patients)
DEMOGRAPHICS/MEDICAL HISTORY	X									
REGISTRATION	X									
RANDOMISATION (INTERVENTION AND CONTROL ARMS ONLY)	X									
ROCKET PLEURAL VENT INSERTION OR ASPIRATION/CHEST DRAIN INSERTION		X								
DAILY OBSERVATIONS (HR, RESPIRATORY RATE, OXYGEN REQUIREMENT, BP, CHEST DRAIN/DEVICE SITE ASSESSMENT) WHILST DEVICE/DRAIN IN SITU			X							
TRIAL BLOOD SAMPLE (5MLS)	X (Day 0 or 1)									
TREATMENT INFORMATION						X				
SAFETY DATA COLLECTION						X	X	X	X	X
DIGITAL AIR LEAK MEASUREMENT (THOPAZ+ DEVICE)			X							
SURGICAL REFERRAL REVIEW					X					
CHEST X-RAY			X	X			X	X	X	X
CT SCANNING							X (1-2 weeks post completion of treatment)			
VAS ASSESSMENT (PAIN AND BREATHLESSNESS)	X		X			X	X	X	X	X
QUALITY OF LIFE ASSESSMENTS (EQ-5D-5L)	X					X	X	X	X	X
PATIENT DIARY (RESOURCE LOG)							X	X	X	X
HEALTH SERVICE UTILISATION/ECONOMICS QUESTIONNAIRES						X	X	X	X	X

[shaded box] Applies to patients in the randomised control trial (intervention and control arms) **and** the observational cohort study

© Copyright: The University of Oxford and Oxford University Hospitals NHS Trust 2014

19. APPENDIX C: AMENDMENT HISTORY

Amendment No.	Protocol Version No.	Date issued	Author(s) of changes	Details of Changes made
N/A	2.0	19May2015	Dr Rob Hallifax/Magda Laskawiec-Szkonter	Inclusion/exclusion criteria corrected (p.12).
Minor Amendment 1	3.0	19Jun2015	Dr Rob Hallifax	Clarification regarding the interventions and X-rays processes, (pp. 20-21, and flowchart p. 35).
Substantial Amendment SA02	4.0	24Aug2015	Dr Rob Hallifax	1. Update CT scan details (Section 7.7)<br>2. Clarify enrolment of Observation group: how to contact and how late can be enrolled.<br>3. Correction of observational cohort study schedule.
Substantial Amendment SA03	5.0	17Nov2015	Dr Rob Hallifax/Magda Laskawiec-Szkonter	1. Review inclusion age to ≥16 years old.<br>2. Clarify that patients enrolled in the Observational cohort study can be subsequently invited to take part in the treatment part of the study if they require intervention.<br>3. Clarification to follow-up schedule and corrections to the flowchart.
Substantial Amendment SA05	6.0	27Apr2016	Dr Rob Hallifax/Magda Laskawiec-Szkonter	1. Changes to the safety section (removal of the need to report recurrence of pneumothorax as an SAE).<br>2. Removal of the “up to 1 week post completion of treatment” time point for SAE recording/reporting and extension of relevant AEs/SAEs recording throughout the trial.<br>3. Addition of recurrence of pneumothorax and

© Copyright: The University of Oxford and Oxford University Hospitals NHS Trust 2014

				re-expansion pulmonary oedema as expected AEs.<br>4. Clarification that "Discharge criteria" are to be used for research purposes only.<br>5. Addition of a new recruiting site: King's College Hospital NHS Foundation Trust.
SA12	7.0	17Nov2016	Dr Rob Hallifax	1. Removal of the need to measure air leak for up to 1 hour.<br>2. Changing the target for the observational cohort from 50 to no upper limit.<br>3. Clarification that childhood asthma is not an exclusion criterion.
SA14	8.0	03Nov2017	Dr Rob Hallifax	Addition of interim analysis of secondary outcomes (blinded date) for the purpose of Dr Hallifax's PhD.

© Copyright: The University of Oxford and Oxford University Hospitals NHS Trust 2014
